# Phenolamide Extract of Apricot Bee Pollen Alleviates DSS-Induced Ulcerative Colitis in Mice by Reducing Oxidative Stress, Modulating Inflammation, and Regulating Gut Microbiota

**DOI:** 10.3390/antiox15030403

**Published:** 2026-03-23

**Authors:** Wei Liu, Rui Liu, Yihang Han, Xin Chen, Qun Lu

**Affiliations:** 1 College of Food Science and Technology, Huazhong Agricultural University, Wuhan 430070, China; liuwei_lw@webmail.hzau.edu.cn (W.L.); liurui@mail.hzau.edu.cn (R.L.); hyu852815@gmail.com (Y.H.); alexischristopher531065@gmail.com (X.C.); 2 Wuhan Engineering Research Center of Bee products on Quality and Safety Control, Wuhan 430070, China; 3 Key Laboratory of Environment Correlative Dietology (Huazhong Agricultural University), Ministry of Education, Wuhan 430070, China

**Keywords:** bee pollen, phenolamide, ulcerative colitis, gut microbiota, short-chain fatty acid

## Abstract

Phenolamides in bee pollen exhibit notable bioactivities, such as antioxidant, anti-inflammatory, and antimicrobial effects. Ulcerative colitis (UC) is a prevalent intestinal disorder, while the potential effects of phenolamides on UC remain unclear. This study aims to investigate the effects and mechanisms of phenolamide extract (PAE) from apricot bee pollen on dextran sulfate sodium (DSS)-induced UC in mice. Firstly, we analyzed the main compounds of PAE. Mice were treated with PAE (100, 200, and 400 mg/kg bw) both during the 7 days preceding 2.5% DSS induction and throughout the induction period (7 days). The results show that the primary compounds of PAE were isomers of tri-*p*-coumaroyl spermidine (97.78 ± 2.76%). A biochemical analysis showed that PAE decreased the levels of pro-inflammatory cytokines and increased the activities of antioxidant enzymes. Regarding the gut microbiota, PAE reduced the Bacillota/Bacteroidota ratio. Additionally, PAE elevated beneficial bacteria, including *norank_f*_*Muribaculaceae*, *norank_o*_*Clostridia*_*UCG-014*, and *Lachnospiraceae*_*NK4A136*_*group*, while reducing harmful bacteria, including *Escherichia-Shigella*, *Clostridium*, and *Romboutsia.* A quantitative analysis of short-chain fatty acids (SCFAs) demonstrated that PAE intervention promotes the biosynthesis of SCFAs in UC mice. This study first demonstrates that PAE attenuates DSS-induced colitis by modulating gut microbiota and SCFAs, suggesting its potential as a functional dietary supplement for colitis.

## 1. Introduction

Inflammatory bowel disease (IBD), as a common chronic intestinal disease, severely compromises patients’ quality of life [1]. Over the previous few decades, IBD has rapidly spread worldwide, with its overall incidence showing a persistent upward trend [2]. Ulcerative colitis (UC) is a main subtype of IBD, characterized pathologically by chronic recurrent inflammation of the colonic mucosa [1]. The pathogenesis of UC is highly complex, resulting from the combined effects of multiple factors, including imbalance of the gut microbiota and its metabolites, oxidative stress, immune dysregulation, and compromised intestinal barrier integrity [3]. The current clinical treatment options for UC remain limited and face numerous challenges. Mainstream UC therapies include anti-inflammatory drugs, immunosuppressants, and biologics [4]. However, these drugs have treatment limitations, often causing adverse liver reactions and even leading to complications [4]. Therefore, there is an urgent need to explore alternative treatment options that are both effective and safe for UC patients.

Natural products may serve as a cyclical alternative treatment for UC due to their numerous health benefits, high safety, and minimal side effects. Previous studies have confirmed that various natural products show promising potential when applied to treat or alleviate colitis in mice, such as rhubarb [5], algae [6], raw Pu-erh tea [7], peanut skin [8], and purslane [9] extracts. Apitherapy is a traditional therapeutic practice with a long history involving the use of bee products (propolis, royal jelly, bee pollen, and bee venom) to treat certain diseases. Centuries ago, bee pollen was used to treat burn wounds and gastrointestinal disorders due to its excellent bioactivities [10]. Bee pollen is a pollen formed when bees collect plant pollen and mix it with nectar and their own secretions [11]. It is famous for its rich nutritional content and is called a “miniature nutritional treasure trove” [11]. It contains both the six essential nutrients required by the human body and various bioactive compounds, such as phenolic acids, flavonoids, and phenolamides [12]. The diverse bioactive compounds contained in bee pollen endowed its broad range of bioactivities, including antioxidant [13], anti-inflammatory [14], anti-microbial [15], alleviating alcohol-induced liver injury [16], anti-obesity [17], and other bioactivities. Among these components, phenolamide, also known as hydroxycinnamic acid amide, is an important active component in bee pollen [18]. Phenolamides are formed by coupling hydroxycinnamic acids (*p*-coumaric acid, ferulic acid, and caffeic acid) with aliphatic amines (putrescine, spermidine, and spermine) via amide bonds [19]. The diverse combinations and degrees of substitution between these hydroxycinnamic acids and aliphatic amines confer a high degree of structural diversity upon phenolamides [20]. Previous studies indicated that bee pollen is rich in phenolamides compared to other natural products, making it an important source of phenolamides [12,20]. However, the previous research primarily focused on phenolamide compounds in relation to plant growth and development, as well as their role in resisting external stresses, with less attention paid to the health benefits of phenolamides. In recent years, research on the health effects of phenolamides has only gradually begun to emerge. It has been discovered that phenolamide possesses bioactivities, such as antioxidant, anti-inflammatory, and anti-alcoholic liver damage properties [16,21]. Nonetheless, it remains unclear whether the phenolamides in bee pollen exert a positive regulatory effect on UC.

Previous studies have consistently demonstrated that UC is closely associated with gut dysbiosis and alterations in the abundance of specific microorganisms [22,23]. Patients with UC exhibit reduced gut microbiota diversity, characterized by an increased Bacillota/Bacteroidota ratio, alongside elevated levels of Proteobacteria [24]. Thus, modulating the gut microbiota may represent a key mechanism by which phenolamides alleviate colitis symptoms. Simultaneously, when gut bacteria break down indigestible food components like polyphenols and dietary fiber, they produce short-chain fatty acids (SCFAs)—important metabolic byproducts of the gut [25]. SCFAs refer to saturated fatty acids with a carbon chain length of less than six carbon atoms, exhibiting multiple physiological functions in maintaining gut health [26]. For instance, SCFAs provide energy to intestinal epithelial cells and help to maintain normal intestinal barrier function [25]. Furthermore, as signaling molecules, SCFAs participate in immune regulation and suppress inflammatory responses [26]. Previous studies have confirmed that polyphenols alleviate UC by promoting SCFA biosynthesis. Taladrid et al. [27] conducted in vitro dynamic gastrointestinal and colonic digestion experiments with grape skin extract that is rich in polyphenols and concluded that colonic digestive fluids were rich in SCFAs and provided more effective intestinal barrier protection compared to gastrointestinal digestion fluids. Huang et al. [28] found that lychee pulp polyphenols promote the biosynthesis of SCFAs (primarily propanoic acid, valeric acid, and isovaleric acid) in the intestines of DSS-induced colitis mice. Phenolamides possess the structural characteristics of polyphenols. Therefore, we hypothesize that phenolamides may improve UC by regulating gut microbiota and SCFAs.

Our preliminary research has demonstrated that the content of phenolamides in apricot bee pollen is relatively high [18]. Based on this, this study utilized apricot bee pollen as the raw material to obtain phenolamide extract (PAE). The composition of PAE was subsequently analyzed by high-performance liquid chromatography with electrospray ionization and quadrupole time-of-flight mass spectrometry (HPLC–ESI–QTOF-MS/MS). Subsequently, mice were administered different doses of PAE (100, 200, and 400 mg/kg bw) via oral gavage prior to dextran sulfate sodium (DSS)-induced colitis, followed by concurrent administration of PAE during the DSS modeling period. We systematically evaluated the effects of PAE intervention on body weight change, feed and water intake, oxidative stress and inflammation status, and colonic morphological changes in DSS-induced colitis mice. Additionally, this study employed 16S rRNA gene sequencing and gas chromatography–mass spectrometry (GC–MS) to further investigate the regulatory effects of PAE on the gut microbiota composition and SCFA content in the cecal contents of DSS-induced colitis mice. This study will provide a theoretical basis for the protective effect of phenolamides against colitis and support their further development in the fields of dietary supplements and functional foods.

## 2. Materials and Methods

### 2.1. Materials

Apricot bee pollen was provided by local beekeepers in Qinglong Manchu Autonomous County, Hebei Province. *N*^1^, *N*^5^, *N*^10^-(*E*)-tri-*p*-coumaroyl spermidine was prepared by our laboratory, and its purity was determined to be 99.01% by peak area normalization using HPLC-DAD. DSS (molecular weight 36–50 kDa) was purchased from MP Biomedicals (Santa Ana, CA, USA). Acetic acid (purity 99.7%) and propanoic acid (purity 99.5%) were purchased from Shanghai Wokai Biotechnology Co., Ltd. (Shanghai, China). Butanoic acid, isobutyric acid, valeric acid, isovaleric acid, hexanoic acid, isohexanoic acid, and the internal standard 2-ethylbutyric acid (all with purity 99%) were obtained from Sigma-Aldrich (MA, USA). HPLC-grade or MS-grade methanol and formic acid were purchased from Thermo Fisher Scientific (Waltham, MA, USA). All other chemicals were analytical-grade reagents purchased from Sinopharm Chemical Reagent Co., Ltd. (Shanghai, China).

### 2.2. Sample Preparation

The preparation of PAE employed a previously established procedure [18] with minor modifications. Firstly, apricot bee pollen (500 g) was subjected to degreasing by sonication at 25 °C for 30 min. This process was repeated twice. The precipitate was extracted twice by sonication with 5 L of 80% ethanol at 25 °C for 30 min. The mixture was vacuum-filtered, and the combined filtrates were concentrated under reduced pressure. The concentrate was diluted with distilled water and then extracted with an equal volume of ethyl acetate. The resulting ethyl acetate phase was concentrated and freeze-dried to obtain a crude extract (4.5 g). The crude extract was loaded onto a silica gel column (Φ5 cm × 48 cm) and eluted sequentially with 100 mL of each of methanol and chloroform mixtures at ratios of 0:100, 5:95, and 10:90. Finally, elution was performed using 1 L of methanol–chloroform mixture (15:85). The fraction eluted under this final ratio was collected as the target fraction. The target fraction was concentrated under reduced pressure and freeze-dried to obtain PAE (2.8 g). It was stored at −20 °C in the dark for subsequent experiments.

### 2.3. Composition Analysis of PAE

The identification and quantification of compounds in PAE were conducted using HPLC–ESI–QTOF-MS/MS and HPLC. For the HPLC, separation was achieved on a Hypersil GOLD C18 column (250 mm × 4.6 mm, 5 μm) with a mobile phase consisting of 0.13% formic acid (A) and methanol (B). The injection volume was 10 μL, with a flow rate of 0.5 mL/min. The gradient elution program was set as follows: 0 min, 50%B; 20 min, 63%B; 22 min, 50%B; 30 min, 50%B, with detection at 280 nm. *N*^1^, *N*^5^, *N*^10^-(*E*)-tri-*p*-coumaroyl spermidine standard was employed to establish a calibration curve for the quantitative analysis of the main compounds in PAE. The separation conditions for the HPLC–ESI–QTOF-MS/MS analysis were identical to those described above. Mass spectrometric detection was performed using Accurate-Mass Q-TOF LC/MS 6520 (Agilent Technologies, Santa Clara, CA, USA) in positive mode; the ionization source was an electrospray ionization (ESI) source with an ionization voltage of 20–40 V. The mass-to-charge ratio (*m*/*z*) scan range was 100–1500, the nebulizer gas pressure was maintained at 30–50 psi, the dry gas flow rate was 8–10 L/min, and the dry gas temperature was 350 °C. The capillary voltage was 3500 V.

### 2.4. Animal Experiment Design

Forty male C57BL/6 mice (22–24 g) were purchased from the Experimental Animal Center of Huazhong Agricultural University (Permission No. SYXK (E) 2020-0084). All animals were housed under specific pathogen-free (SPF) conditions with a 12 h light/dark cycle. The environmental temperature was maintained at 22 ± 2 °C, with a relative humidity of 50–60%. After a 7-day adaptation period, the mice were randomly assigned into five groups (*n* = 8), including the normal control group (NC), the model group (DSS), the low-dose group (DSS + L_PAE), the medium-dose group (DSS + M_PAE), and the high-dose group (DSS + H_PAE). The DSS + L_PAE group, DSS + M_PAE group, and DSS + H_PAE group were given 100 mg/kg bw, 200 mg/kg bw, and 400 mg/kg bw of PAE dissolved in 0.1% CMC-Na by gavage daily for 14 days, respectively. The NC group and the DSS group were given the equal volume of 0.1% CMC-Na by gavage daily at the same time. From the eighth day, except the NC group, all the other groups were allowed free access to a 2.5% (*w*/*v*) DSS solution dissolved in DSS-specific solvent (MA0650, Dalian Meilun Biotechnology Co., Ltd., Dalian, China) for seven days to induce colitis. Mice in the NC group were given the same DSS-specific solvent without DSS. The animal grouping and the corresponding treatment were conducted according to the procedures presented in Figure S1 (Supplementary Materials). The body weight, feed intake, water intake, and fecal consistency of the mice were recorded daily. Subsequently, the mice underwent a 12-hour fasting period, during which they had free access to water. After the fasting period, all animals were anesthetized via isoflurane inhalation, and blood samples were collected from the retro-orbital venous plexus, followed by cervical dislocation. The cecal contents, colon, spleen, liver and kidneys of the mice were immediately excised. The colon length, as well as the weights of the spleen, liver, and kidneys, were recorded on-site. A fecal-free colon segment approximately 1 cm in length was stored in neutral tissue fixative (Wuhan Servicebio Technology Co., Ltd., Wuhan, China). The cecal contents were placed in 2 mL sterile cryovials; remaining colon tissues were sealed in zip-lock bags, and all samples were subsequently stored at −80 °C. All experimental procedures and animal welfare management were conducted according to the Guidelines for the Care and Use of Experimental Animals: 8th edition (ISBN-10: 0-309-15396-4).

### 2.5. Macroscopic Evaluation

During the modeling period, the survival status, body weight, feed intake, and water intake of the mice were monitored daily. Daily feed intake and water intake were determined by weighing the bottles and feed. The mice underwent daily abdominal massage to stimulate defecation, and the consistency of their feces was observed. Subsequently, the severity of fecal occult blood in mice was determined using a human fecal occult blood test kit (Nanjing Jiancheng Bioengineering Institute, Nanjing, China). According to Table S1 (Supplementary Materials), the scores of the body weight loss, stool consistency and fecal occult blood of each mouse were scored. Finally, the disease activity index (DAI) was calculated as previously reported [29], which was estimated as the average score of the body weight loss, stool consistency, and fecal occult blood. The organ indices (including spleen, liver and kidneys) were calculated according to the following formula: organ index = organ weight (mg)/body weight (g).

### 2.6. Histological and Alcian Blue Staining of Colon Tissue

H&E staining was employed to assess the severity of colonic injury. The colons of mice that have been fixed for 24 h were paraffin-embedded, sectioned into 4 μm slices, and subjected to stepwise xylene-based dewaxing. The sections were then stained with hematoxylin and eosin solutions, dehydrated using a gradient ethanol series, and mounted with neutral resin. Colon structure, crypts, mucosal layers, and inflammatory infiltration were evaluated by examining the sections under 40×, and 100× magnification using an NB610 optical microscope (Ningbo Yongxin Optics Co., Ltd., Ningbo, China). Histology score was determined according to a previous report [8]. The scored standard is shown in Table S2 (Supplementary Materials), with the total score derived from the sum of the infiltration and mucosal damage subscores.

Mucin and goblet cells were visualized using the AB-PAS staining method. AB-PAS staining was performed following a similar protocol described above using alcian blue solution, periodate solution and Schiff’s reagent. Blue-stained cells represent acidic goblet cells secreting acidic mucin. The number of goblet cells was measured using Image J software (version 1.54g, NIH, Bethesda, MD, USA) and normalized by the measured area.

### 2.7. Determination of Oxidative Stress Indicators in Colon of Mice

Exactly 100 mg of colon was precisely weighed and placed into a 2 mL grinding tube. Subsequently, 0.9 mL of normal saline was added at a ratio of 1:9 (*w*:*v*). The mixture was homogenized and then centrifuged at 10,000× *g* for 10 min at 4 °C. The supernatant was collected for the determination of malondialdehyde (MDA), glutathione peroxidase (GSH-Px), superoxide dismutase (SOD) and catalase (CAT) levels. The above indicators were measured according to the protocols specified in the assay kits using Multiskan SkyHigh microplate reader (Thermo Fisher Scientific, Waltham, MA, USA). These kits were purchased from Nanjing Jiancheng Bioengineering Institute (Nanjing, China). The protein content of supernatant was determined using the bicinchoninic acid (BCA) kit (Beijing Labjic Technology Co., Ltd., Beijing, China). The results are presented using protein content for normalization.

### 2.8. Determination of Inflammatory Indicators of Mice

Blood samples were centrifuged at 3000× *g* for 10 min at 4 °C to obtain serum. The serum was then collected for the analysis of inflammatory cytokine levels. The levels of tumor necrosis factor (TNF)-α, interleukin (IL)-6, IL-1β, IL-10 and lipopolysaccharide (LPS) in the serum were measured using enzyme-linked immunosorbent assay (ELISA) kits from Shanghai Hengyuan Biotechnology Co., Ltd. (Shanghai, China). The level of myeloperoxidase (MPO) in colon tissue was determined using a biochemical assay kit from Nanjing Jiancheng Bioengineering Institute (Nanjing, China).

### 2.9. Analysis of Gut Microbiota

The 16S rRNA sequencing analysis of the cecal microbiota was performed using a previously reported method [30]. Firstly, genomic DNA was extracted from cecal contents. The V3–V4 region of the 16S rRNA gene was amplified by PCR with the forward primer 338F (5′-ACTCCTACGGGAGGCAGCAG-3′) and reverse primer 806R (5′-GGACTACHVGGGTWTCTAAT-3′). Subsequently, PCR products were purified and sequenced according to the standard operating procedures of MajorBio Biotechnology Co., Ltd. (Shanghai, China) on the Illumina NextSeq 2000 platform (Illumina, SD, USA). After sequencing, α-diversity, β-diversity analysis and other bioinformatics analyses of the gut microbiota were performed using the MajorBio Cloud platform (https://cloud.majorbio.com (assessed on 28 July 2025)).

### 2.10. Determination of SCFAs in Cecal Contents

The measurement of SCFA contents was carried out as previously described [31] with minor modification. Cecal contents were homogenized with 0.5% phosphoric acid solution, followed by centrifugation to separate the liquid and solid phases. The supernatant was obtained for analysis, with 10 μg/mL 2-ethylbutyric acid added as an internal standard. SCFAs were quantified using an Agilent 8890B gas chromatograph coupled with an Agilent 7000D mass spectrometer detector equipped with an Agilent HP-FFAP capillary column (30 m × 0.25 mm, 0.25 μm) (Santa Clara, CA, USA). The injection volume was 1 μL, with a split ratio of 10:1. The content of SCFAs was calculated by standard calibration curves. The total content of SCFAs was calculated by summing the quantities of all detected SCFAs.

### 2.11. Statistical Analysis

Data are presented as the mean ± standard error of mean (SEM). One-way analysis of variance (ANOVA) followed by Duncan’s test were performed by SPSS statistics 26 software (IBM, Chicago, USA) to access statistical significance. *p*-value of less than 0.05 was considered statistically significant in this study. Spearman correlation coefficients were calculated using the MajorBio Cloud platform (http://cloud.majorbio.com (assessed on 23 August 2025)). The correlation network diagram was generated using Cytoscape (version 3.9.1, https://cytoscape.org (assessed on 22 December 2025)).

## 3. Results and Discussion

### 3.1. Composition of PAE

**Figure 1:**
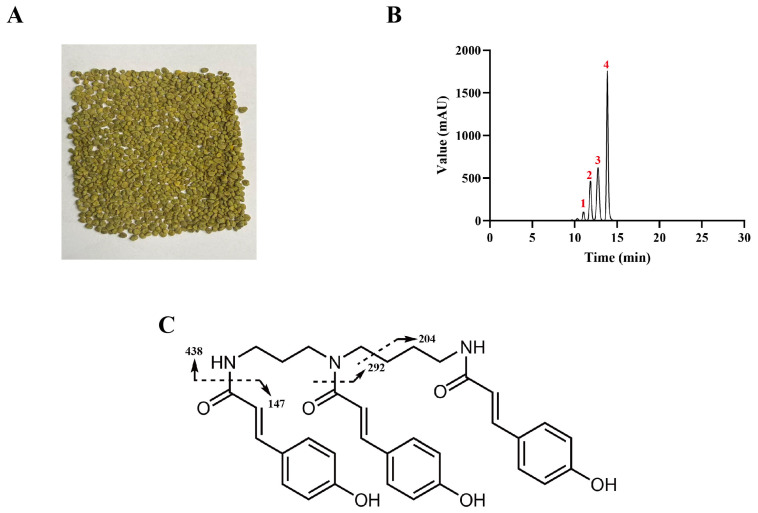
(**A**) Apricot bee pollen; (**B**) HPLC chromatograph of PAE; (**C**) mass spectrometry fragmentation of main compounds in PAE. Peaks 1–4 mean the main compounds in PAE. Note: The arrows indicate the sites of fragmentation.

The main compounds in PAE were identified using HPLC–ESI–QTOF-MS/MS, and their quantitative analysis was performed via HPLC. The representative image of apricot bee pollen is shown in Figure 1A. The HPLC chromatogram indicates that the main compounds of PAE correspond to the compounds represented by peaks 1–4 (Figure 1B). The retention times, MS data, and purities for these compounds corresponding to peaks 1–4 are shown in Table 1. Peaks 1–4 exhibit the same molecular ion peak (*m*/*z* 584) and identical characteristic fragment ion peaks (*m*/*z* 147, *m*/*z* 204, *m*/*z* 292, and *m*/*z* 438), suggesting that these are probably isomers. The different retention times of these four isomers indicate differences in their polarity [32]. Phenolamides are typically formed by amide linkages between polyamines, such as putrescine (C_4_H_12_N_2_), spermidine (C_7_H_19_N_3_), and spermine (C_10_H_26_N_4_), and hydroxycinnamic acids. The latter are commonly *p*-coumaric acid (C_9_H_8_O_3_), caffeic acid (C_9_H_8_O_4_), and ferulic acid (C_10_H_10_O_4_). Therefore, the type of polyamine and the number of substituents can be approximately deduced from the predicted molecular formula. The MS fragmentation pattern is illustrated in Figure 1C. The peak at *m*/*z* 438 corresponds to the loss of a *p*-coumaroyl group. The peak at *m*/*z* 292 indicates the subsequent loss of a second *p*-coumaroyl group. The peak at *m*/*z* 204 results from the cleavage of a combination of three alkyl groups and a *p*-coumaroyl residue, and the peak at *m*/*z* 147 represents a *p*-coumaroyl residue. The deduced fragmentation pathways are consistent with a supporting report [12]. The *E*/*Z* configuration isomerism in the phenylpropane fraction and hindered rotation of *N*^5^-amide bond lead to various phenolamide isomers [17]. Therefore, peaks 1–4 were identified as different isomers of tri-*p*-coumaroyl spermidine. Subsequently, we employed HPLC with *N*^1^, *N*^5^, *N*^10^-(*E*)-tri-*p*-coumaroyl spermidine as a standard to establish a calibration curve (y = 1.4802 x − 9.1717, R^2^ = 0.9998, 1–200 μg/mL) for external standard quantification. The results indicate that the content of tri-*p*-coumaroyl spermidine in PAE was 97.78 ± 2.76% (Table 1).

### 3.2. Effect of PAE on Survival Status, Feed Intake and Water Intake

**Figure 2:**
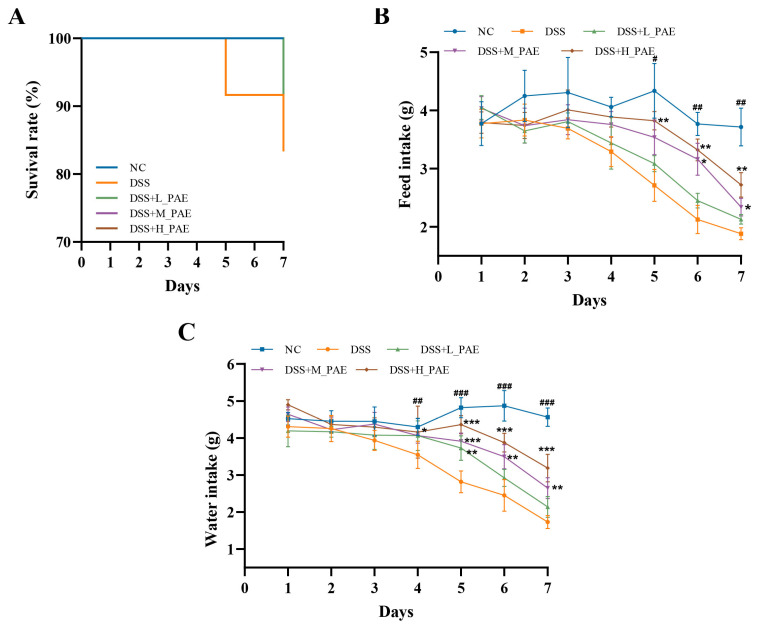
Effects of PAE on pathological symptoms in DSS-induced colitis mice. (**A**) Survival status; (**B**) feed intake; (**C**) water intake. Data are presented as the mean ± SEM. Analysis was carried out using one-way analysis of variance (ANOVA) (compared with NC group, # *p* < 0.05, ## *p* < 0.01, ### *p* < 0.001; compared with DSS group, * *p* < 0.05, ** *p* < 0.01, *** *p* < 0.001).

Two mice in the DSS group and one in the DSS + L_PAE group died during the DSS treatment period, while there were no deaths in the other groups (Figure 2A). One possible explanation is organic damage to the intestine caused by DSS-induced pain. When this pain exceeded the tolerance threshold of the mice, it may have led to their death [33]. In contrast, PAE intervention enhanced the ability of the intestine to resist damage, thereby alleviating discomfort in the DSS-induced colitis mice. Feed intake and water intake are key indicators for evaluating the success of UC model establishment [34]. As observed in Figure 2B,C, feed intake and water intake were significantly reduced in the DSS group compared to the NC group (*p* < 0.05). The primary reason may be that DSS, as a chemical inducer, disrupts the intestinal barrier and causes irritation and injury to the gastrointestinal tract. The resulting severe UC, accompanied by pain and gastrointestinal discomfort, subsequently suppressed the appetite of the mice. Under such conditions, excessive feed intake would further burden the digestive system. In contrast, both feed intake and water intake in mice were partially recovered following PAE intervention. On day 7 of the experiment, feed intake in the PAE-treated groups increased to 1.13-fold (DSS + L_PAE group), 1.24-fold (DSS + M_PAE group), and 1.84-fold (DSS + H_PAE group) that of the DSS group. This beneficial effect may be related to the ability of PAE to alleviate inflammation and associated pain responses caused by DSS. PAE intervention would partially counteract the suppression of feeding and drinking behaviors caused by physiological discomfort in DSS-induced colitis mice.

### 3.3. Effect of PAE on Body Weight and DAI Score

**Figure 3:**
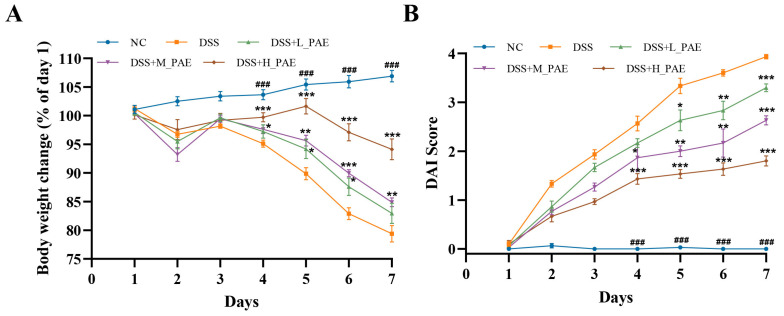
Effects of PAE on body weight change and DAI score in DSS-induced colitis mice. (**A**) Body weight change rate; (**B**) DAI score. Data are expressed as mean ± SEM. Analysis was carried out using one-way analysis of variance (ANOVA) (compared with NC group, ### *p* < 0.001; compared with DSS group, * *p* < 0.05, ** *p* < 0.01, *** *p* < 0.001).

Patients with UC often exhibit typical clinical symptoms, such as body weight loss, loose stools, and fecal blood [7]. The changes in body weight across the groups are shown in Figure 3A. Compared to the NC group, the mice in the DSS group exhibited a significant decrease in body weight due to continuous intake of drinking water containing 2.5% DSS for 7 days (*p* < 0.05). By day 7 of modeling, the body weight of the DSS group mice had decreased to 78.37% of that on day 1. This phenomenon may be attributed to DSS-induced gastrointestinal discomfort leading to anorexia, resulting in insufficient energy intake, while malabsorption caused by intestinal villus damage collectively contributes to body weight loss [35]. Moreover, body weight began to decline sharply starting from day 4 after DSS treatment. Following intervention with different doses of PAE, PAE significantly delayed rapid body weight loss in the DSS-induced colitis mice (*p* < 0.05). A possible reason is that PAE increased the feed intake of the mice and partially restored their intestinal nutrient absorption capacity, thereby enhancing its energy replenishment effect.

The DAI score is a comprehensive assessment score based on three pathological indicators of UC: body weight loss, fecal blood, and loose stools. It reflects the severity of disease progression [36]. To evaluate the pathological symptoms of colitis in mice comprehensively, body weight changes and fecal status were monitored daily during the modeling period, and the DAI score was calculated accordingly. The DAI score results are presented in Figure 3B. Starting from day 4 of the modeling period, the mice in the DSS group exhibited fecal blood and loose stools. Thus, the DAI score of the DSS group became significantly higher than that in the NC group (*p* < 0.05), indicating the successful establishment of the UC model. The PAE treatment significantly ameliorated this adverse change. On day 7 of modeling, the DAI score of the DSS group reached 3.93. However, PAE intervention at different doses reduced the DAI score by 16.03% (DSS + L_PAE group), 33.08% (DSS + M_PAE group), and 54.20% (DSS + H_PAE group) compared to the DSS group. These results indicate that the PAE intervention effectively alleviated pathological features in DSS-induced colitis mice, including body weight loss, fecal blood, and loose stools.

### 3.4. Effect of PAE on Spleen, Liver and Kidney Indices

**Figure 4:**
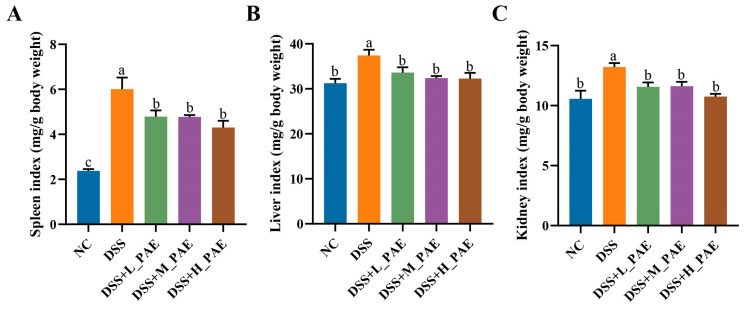
Effects of PAE on organ indices in DSS-induced colitis mice. (**A**) Spleen index; (**B**) liver index; (**C**) kidney index. Data are expressed as mean ± SEM. Mean values with different superscript letters are significantly different (*p* < 0.05) based on one-way analysis of variance (ANOVA) with Duncan’s range test.

The spleen is one of the most important peripheral immune organs in the body. It plays a key role in immune regulation [37]. Under inflammatory conditions, the immune function of the spleen is persistently activated, often accompanied by tissue hyperplasia to promote the secretion of anti-inflammatory factors such as IL-10 [38]. Therefore, the spleen index (the ratio of spleen weight to body weight) is commonly used as a preliminary indicator to assess the degree of systemic inflammation [39]. The spleen index results are shown in Figure 4A. Compared with the NC group, the spleen index of the DSS group mice was significantly increased by 2.53-fold (*p* < 0.05), suggesting that the persistent inflammatory state may have led to pathological enlargement of the spleen. Following intervention with different doses of PAE, the spleen index was significantly reduced compared to the DSS group (*p* < 0.05), decreasing by 20.29% (DSS + L_PAE group), 20.47% (DSS + M_PAE group), and 28.36% (DSS + H_PAE group). This indicates that PAE may alleviate spleen enlargement by attenuating the immune response.

To further evaluate the impact of PAE on inflammation in extra-intestinal organs, this study also examined the liver and kidney indices. The results are shown in Figure 4B,C. Both the liver and kidney indices of the DSS group mice were significantly elevated (*p* < 0.05), suggesting swelling of extra-intestinal tissues. A possible explanation is that, when the intestinal barrier is disrupted, a large number of bacterial-derived harmful products enter the circulatory system and subsequently reach the liver and kidneys via the portal vein [40]. The PAE intervention reversed the swelling of both the liver and kidneys. This indirectly indicates that PAE may help to maintain intestinal barrier integrity and effectively suppress pathological damage to extra-intestinal organs caused by harmful bacterial products from the gut.

### 3.5. Effect of PAE on Oxidative Stress

**Figure 5:**
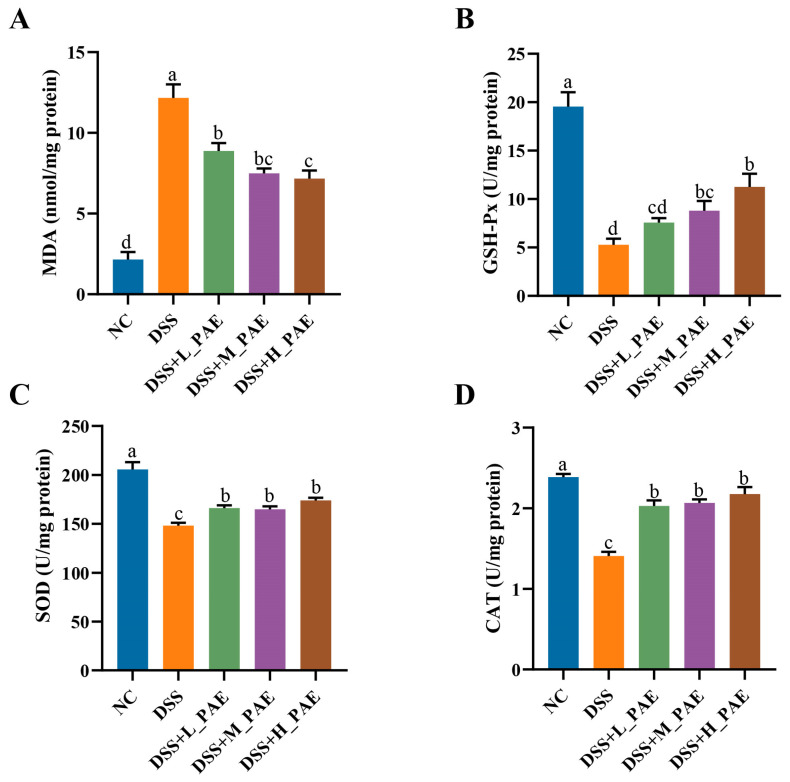
Effects of PAE on colon oxidative stress injury in DSS-induced colitis mice. (**A**) MDA content; (**B**) GSH-Px activity; (**C**) SOD activity; (**D**) CAT activity. Data are expressed as mean ± SEM. Mean values with different superscript letters are significantly different (*p* < 0.05) based on one-way analysis of variance (ANOVA) with Duncan’s range test.

Under normal physiological conditions, a balance exists between reactive oxygen species (ROS) and cellular antioxidant defense capacity. However, oxidative stress occurs when the production of ROS exceeds the ability of the antioxidant defense system to eliminate these toxic components. This leads to cellular structural damage, increased membrane permeability, aggravated inflammatory responses, and other pathological processes [41]. Among the substances that defend against oxidative stress, antioxidants that directly scavenge ROS and block oxidation reactions, together with endogenous antioxidant enzymes, constitute the cellular antioxidant defense system [42]. MDA is one of the primary products of lipid peroxidation, and its concentration is closely associated with the degree of lipid peroxidation [43]. When free radicals react with lipids in the body, lipid peroxidation occurs, ultimately generating MDA. Therefore, MDA levels can indirectly reflect the extent of oxidative stress. Additionally, the antioxidant enzymes GSH-Px, SOD, and CAT play a critical role in scavenging excess free radicals in the body. They are essential components of the endogenous antioxidant defense system. Therefore, their activity levels are closely associated with the severity of UC [44,45]. GSH-Px eliminates hydrogen peroxide by coupling its reduction with the oxidation of glutathione [44]. SOD is a metalloprotein that catalyzes the removal of superoxide radicals, producing hydrogen peroxide as the final reaction product [44]. CAT is a heme-containing enzyme that catalyzes the conversion of hydrogen peroxide (H_2_O_2_) into water (H_2_O) and oxygen (O_2_) [45]. Therefore, measuring the levels of antioxidant enzymes in the body helps to analyze the oxidative stress status. To determine the protective effect of PAE against DSS-induced oxidative stress in the colon, we examined the content of MDA and the activities of multiple antioxidant enzymes. As shown in Figure 5A–D, compared with the NC group, the MDA content in colon tissue of DSS-induced mice was significantly increased (*p* < 0.05), while the activities of GSH-Px, SOD and CAT were significantly decreased (*p* < 0.05), indicating marked oxidative stress injury in the DSS group. The MDA level in the DSS group increased by 42.58% compared to the NC group. After the PAE intervention, the MDA levels in the colon decreased by 16.05% (DSS + L_PAE group), 24.04% (DSS + M_PAE group), and 28.64% (DSS + H_PAE group) relative to the DSS group. The activities of key antioxidant enzymes were enhanced. The GSH-Px activity increased by 43.34% (DSS + L_PAE group), 66.75% (DSS + M_PAE group), and 113.45% (DSS + H_PAE group) compared to the DSS group. The SOD activity was elevated by 12.05% (DSS + L_PAE group), 11.29% (DSS + M_PAE group), and 17.39% (DSS + H_PAE group). The CAT activity rose by 44.09% (DSS + L_PAE group), 46.81% (DSS + M_PAE group), and 54.50% (DSS + H_PAE group). The above results indicate that PAE alleviates oxidative stress injury in the intestinal mucosa of DSS-induced colitis mice by reducing the content of lipid peroxidation products (MDA) and enhancing the activities of key antioxidant enzymes.

### 3.6. Effect of PAE on Inflammatory Status

**Figure 6:**
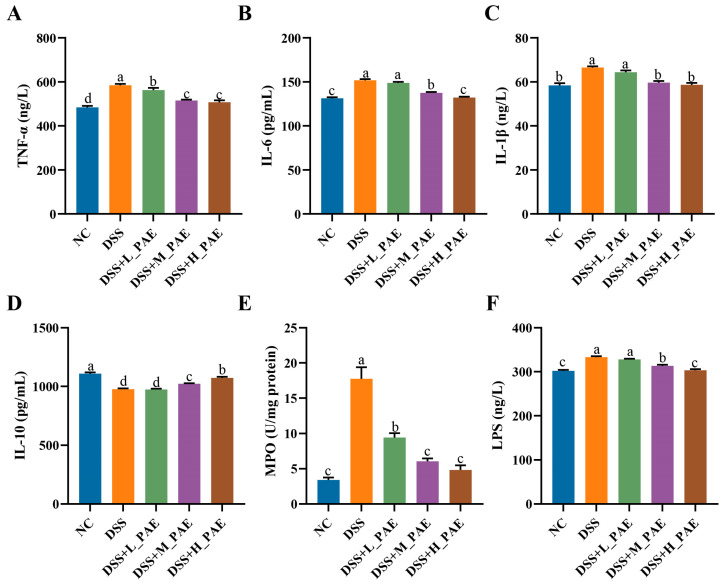
Effects of PAE on serum and colon inflammation in DSS-induced colitis mice. (**A**) TNF-α content in serum; (**B**) IL-6 content in serum; (**C**) IL-1β content in serum; (**D**) IL-10 content in serum; (**E**) MPO activity in colon tissue; (**F**) LPS content in serum. Data are expressed as mean ± SEM. Mean values with different superscript letters are significantly different (*p* < 0.05) based on one-way analysis of variance (ANOVA) with Duncan’s range test.

In chronic diseases such as UC, inflammation and oxidative stress often act synergistically to jointly promote disease progression [46]. Disruption of the intestinal barrier allows gut antigens to be presented to immune cells in the mucosa and submucosa, leading to abnormal activation of these immune cells and subsequent upregulation of inflammatory cytokines. TNF-α and IL-6 have been demonstrated to exacerbate intestinal barrier dysfunction by inducing mucosal damage [47]. Conversely, the expression of the anti-inflammatory factor IL-10 is markedly suppressed. This factor is primarily secreted by macrophages and plays a key role in alleviating inflammation [48]. To assess the effect of PAE on inflammatory cytokine levels in DSS-induced colitis mice, the levels of TNF-α, IL-6, IL-1β and IL-10 were measured in the serum of the mice. The results are shown in Figure 6A–D. Pro-inflammatory cytokines were significantly elevated (*p* < 0.05), while anti-inflammatory cytokines were significantly reduced (*p* < 0.05), indicating that the inflammatory response in the mice was significantly enhanced following DSS induction. In contrast, after the PAE treatment, the levels of pro-inflammatory cytokines decreased significantly (*p* < 0.05). Intervention with different doses of PAE reduced serum TNF-α by 3.81–13.25%, IL-6 by 2.03–12.97%, and IL-1β by 3.20–11.78% compared to the DSS group. Following a high-dose PAE intervention, the serum IL-6 and IL-1β levels in the mice showed no significant difference compared to the NC group (*p* > 0.05). These results demonstrate that PAE helps to maintain the balance between pro-inflammatory and anti-inflammatory cytokines. Additionally, this study measured the activity of MPO in mouse colon tissue and the content of LPS in mouse serum. MPO is primarily released by activated neutrophils. This enzyme promotes oxidative stress and inflammatory damage by catalyzing the production of oxidants, such as hypochlorous acids. Its activity reflects the extent of neutrophil infiltration in tissues [29,49]. The experimental results (Figure 6E) show that PAE significantly reduced MPO activity in mouse colon tissue. Intervention with different doses of PAE decreased colon MPO activity by 46.93–72.81% compared to the DSS group, and no significant differences were observed between the DSS + M_PAE group, DSS + H_PAE group and NC group. These results indicate that PAE can effectively suppress neutrophil accumulation and the associated oxidative damage, thereby alleviating the pathological progression of DSS-induced colitis. LPS is a component of the outer membrane of Gram-negative bacterial cell walls. DSS damages the colon tissue of mice, disrupts the intestinal barrier, and increases barrier permeability. This results in elevated serum LPS levels, subsequently triggering a systemic inflammatory response [50]. The result of LPS content in serum is shown in Figure 6F. Compared with the NC group, the LPS content in the serum of the DSS-treated mice was significantly elevated (*p* < 0.05), indicating intestinal barrier damage in the DSS-induced mice, which allowed LPS translocation into the blood and consequently increased the serum LPS level. However, the PAE intervention partially reversed this change. The DSS + M_PAE group and DSS + H_PAE group showed a significant decrease compared to the DSS group (*p* < 0.05), while no significant difference was observed between the DSS + H_PAE group and NC group (*p* > 0.05). Overall, these findings indicate that PAE may help to maintain intestinal barrier integrity and reduce abnormal immune responses in immune cells, thereby alleviating inflammation in DSS-induced colitis mice.

### 3.7. Effects of PAE on Colonic Tissue Morphology

**Figure 7:**
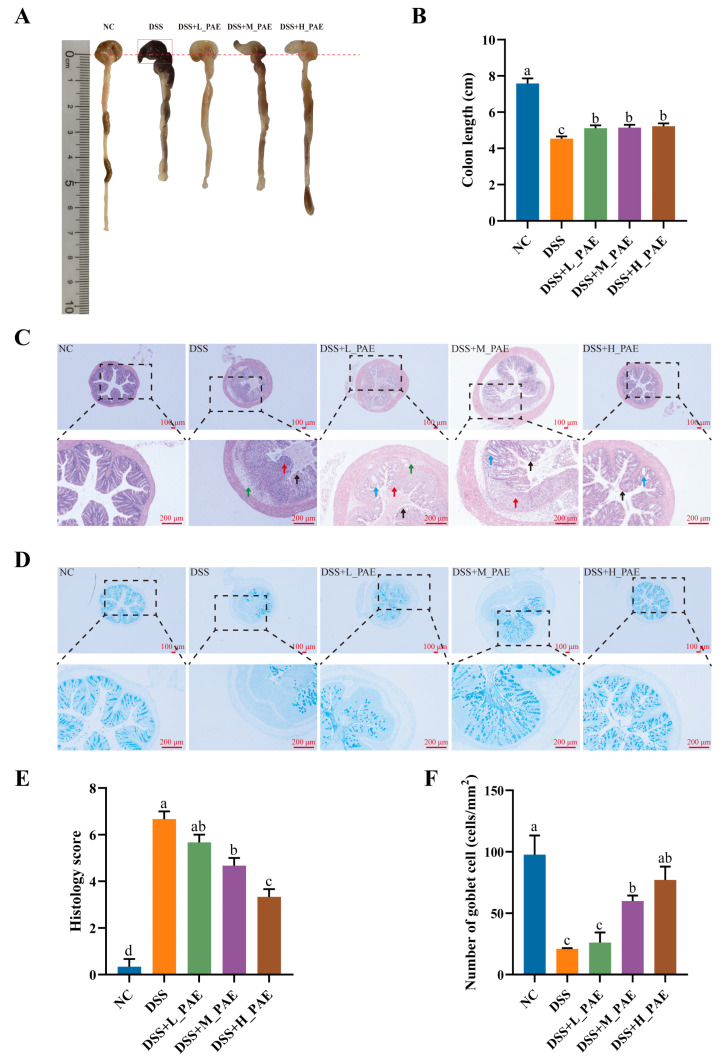
Effects of PAE on colon morphology in DSS-induced colitis mice. (**A**) Representative images of colon tissue; (**B**) colon length; (**C**) H&E-stained section images (40×, 100×); (**D**) AB-PAS-stained section images (40×, 100×); (**E**) histology score; (**F**) goblet cell count. Data are expressed as mean ± SEM. Mean values with different superscript letters are significantly different (*p* < 0.05) based on one-way analysis of variance (ANOVA) with Duncan’s range test. The red arrows indicate inflammatory cell infiltration, the green arrows represent mucosal edema, the blue arrows point to partial crypt restoration, and the black arrows indicate blurred contours and disordered glandular structures.

Colon shortening is a typical symptom in UC patients, and colon length serves as an important reference index for UC [7]. Representative colon photographs from each group are shown in Figure 7A. The red boxes in the image visually indicate that the DSS-induced mice exhibited significant swelling at the cecal end, ulceration, and a large amount of bloody feces. These symptoms were alleviated following PAE administration. Moreover, the result for colon length is shown in Figure 7B. The average colon length of the DSS-induced mice was 4.54 cm, which was significantly shorter than the 7.58 cm of the NC group (*p* < 0.05). By comparison, the colon lengths of the treatment groups were significantly increased (*p* < 0.05). In summary, PAE significantly alleviated colonic swelling and atrophy. To further investigate the pathological damage induced by DSS in mouse colon, a histopathological evaluation was performed on transverse sections of mouse colon tissue, and the detailed information is shown in Table S2 (Supplementary Materials). The representative images of H&E staining and AB-PAS staining analysis are shown in Figure 7C and Figure 7D, respectively. The colon tissues of the DSS-induced mice exhibited severe submucosal edema, thickened intestinal walls, neutrophil infiltration, and disappearance of most crypts with blurred contours and disordered glandular structures. Following the PAE treatment, the boundaries and contours of mouse colon tissue became clearer, crypts showed partial recovery, and mucosal edema was improved. The histology score could comprehensively evaluate the severity of intestinal damage and inflammation. The histology scores in the PAE intervention group were reduced by 15%, 17.65%, and 28.57% compared to the DSS group (Figure 7E). The mucus layer is composed of a mesh-like MUC2 mucin secreted by goblet cells, which binds to Alcian blue and appears blue in staining [51]. The number of goblet cells in the DSS group was significantly lower than that in the NC group (*p* < 0.05). The PAE treatment significantly improved this adverse effect (*p* < 0.05) by increasing the goblet cell count by 1.25-, 2.87-, and 3.69-fold compared to the DSS group (Figure 7F). The above experimental results indicate that, in the DSS-induced mice, mucosal damage, inflammatory cell infiltration, and decreased goblet cell number led to compromised intestinal mucosal integrity. The PAE intervention effectively alleviated these pathological symptoms and may have enhanced mucus secretion, thereby better repairing the intestinal damage in the DSS-induced colitis mice.

### 3.8. Effect of PAE on Gut Microbiota

**Figure 8:**
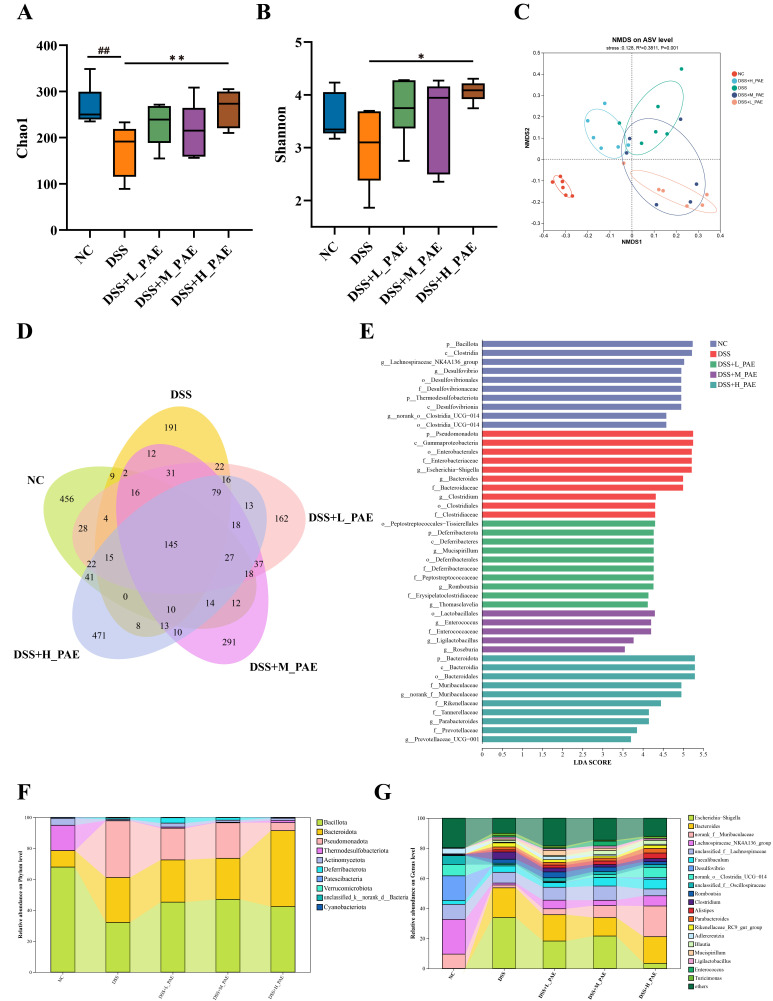
Effects of PAE on gut microbiota in DSS-induced colitis mice. (**A**) Chao1 index; (**B**) Shannon index; (**C**) NMDS analysis; (**D**) Venn plots showing ASVs in gut microbiota among groups; (**E**) linear discriminant analysis with LDA score greater than 3.5; (**F**) relative abundance of representative bacteria at phylum level; (**G**) relative abundance of representative bacteria at the genus level. Data are expressed as mean ± SEM. Analysis was carried out using one-way analysis of variance (ANOVA) (compared with NC group, ## *p* < 0.01; compared with DSS group, * *p* < 0.05, ** *p* < 0.01).

Changes in gut microbial composition are closely associated with the progression of UC [52]. In α-diversity analysis, the Chao1 and Shannon indices are important metrics for assessing the diversity and richness of gut microbiota. Reduced gut microbial diversity and richness are typical features of patients with intestinal diseases [53]. The results of α-diversity are shown in Figure 8A,B. The Chao1 index of the gut microbiota in the DSS group was significantly lower than that in the NC group (*p* < 0.05). The PAE intervention markedly increased both indices, and, particularly under high-dose PAE, the Chao 1 and Shannon indices of the gut microbiota showed significant improvements compared to the DSS group (*p* < 0.05). Thus, DSS induction reduces the richness of gut microbiota in UC mice, an effect that can be modulated by PAE intervention. The β-diversity analysis reveals overall clustering patterns and significant separation among the experimental groups, with intergroup distances reflecting the degree of similarity in gut microbial composition [54]. In this study, non-metric multidimensional scaling (NMDS) analysis based on unweighted-unifrac distances revealed clear separation between the NC group and the other groups. While partial overlap was observed between the DSS group and DSS + M_PAE and DSS + H_PAE groups, they remained distinguishable (Figure 8C). Based on a 99.9% sequence similarity threshold, 1010, 598, 683, 706, and 902 amplicon sequence variants (ASVs) were identified in the NC, DSS, DSS + L_PAE, DSS + M_PAE, and DSS + H_PAE groups, respectively (Figure 8D). Compared with the NC group, the number of ASVs in the DSS group was significantly reduced (*p* < 0.05). The number of ASVs in the PAE intervention groups was significantly increased compared to the DSS group (*p* < 0.05). These results indicate that DSS induction significantly altered the composition of gut microbiota in mice, while the PAE intervention could reverse these adverse changes.

Subsequently, linear discriminant analysis effect size (LEfSe) was employed to further analyze the impact of PAE on the gut microbiota of DSS-induced mice (Figure 8E). The DSS group was predominantly enriched with pathogenic bacteria, including Pseudomonadota, *Enterobacterales*, *Escherichia*-*Shigella*, *Bacteroidaceae*, *Clostridium*, and *Clostridiaceae*. The DSS + L_PAE group was primarily enriched with Deferribacterota, *Deferribacteraceae*, and *Mucispirillum*. The DSS + M_PAE group was primarily enriched with *Lactobacillus* and *Roseburia* while also exhibiting increased proliferation of *Enterococcus* and *Enterococcaceae* family. In the DSS + H_PAE group, significant enrichment was observed in Bacteroidota, *Muribaculaceae*, *norank*_*f*_*Muribaculaceae*, *Rikenellaceae*, *Tannerellaceae*, *Prevotellaceae*, and *Prevotellaceae*_*UCG*-*001*. These results indicate that a medium-dose PAE intervention can partially improve gut microbial composition by increasing the abundance of certain beneficial bacterial. Furthermore, sustained high-dose PAE intake significantly reverses the gut microbial composition, shifting it toward that of healthy mice. Moreover, to more precisely identify the main compositional changes in the microbiota, differences in gut microbial communities were examined at the phylum and genus levels. The relative abundances of gut microbiota at the phylum level and the genus level are shown in Figure 8F and Figure 8G, respectively.

**Figure 9:**
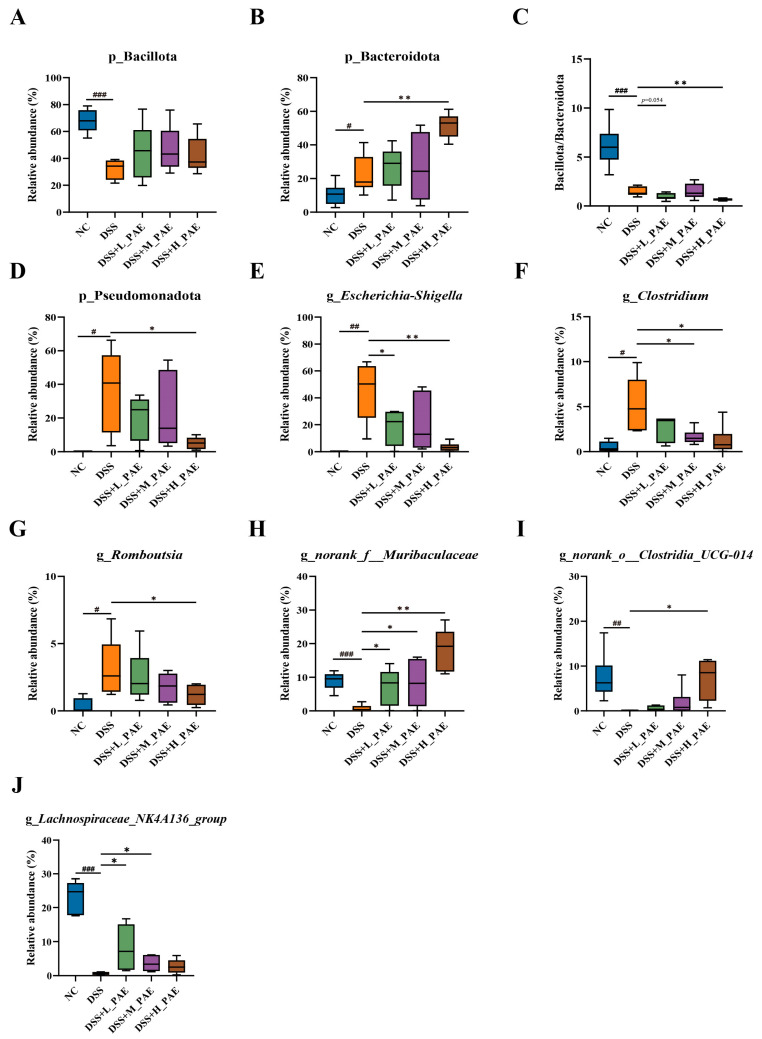
Relative abundance of key bacteria at the phylum and genus levels. Data are expressed as mean ± SEM. (**A**–**D**) Relative abundance of predominant bacteria shown at the phylum level; (**E**–**J**) relative abundance of predominant bacteria shown at genus level. Analysis was carried out using one-way analysis of variance (ANOVA) (compared with NC group, # *p* < 0.05, ## *p* < 0.01, ### *p* < 0.001; compared with DSS group, * *p* < 0.05, ** *p* < 0.01).

At the phylum level, the gut microbiota of mice primarily consisted of Bacillota, Bacteroidota, Pseudomonadota, Actinobacteria, and Deferribacterota. Bacillota and Bacteroidota were dominant, together accounting for over 80% of the total abundance (Figure 9A,B). An elevated ratio of Bacillota and Bacteroidota is an important marker of gut microbiota dysbiosis and may accelerate the progression of colitis [55]. DSS induction resulted in a Bacillota/Bacteroidota ratio of 1.47 ± 0.19 in the gut microbiota of the mice. However, in the DSS + H_PAE group, the Bacillota/Bacteroidota ratio was 0.66 ± 0.05, which was significantly lower than that in the DSS group (*p* < 0.05) (Figure 9C). This indicates that a high-dose PAE intervention can decrease the Bacillota/Bacteroidota ratio in DSS-induced colitis mice. This finding is consistent with previous research [56] and can be partly attributed to the fact that Bacteroidota encompasses a wide range of symbiotic and anti-inflammatory bacterial genera [57]. Pseudomonadota belongs to Proteobacteria and is a common pathogenic bacterium in the gut. Its abundance is higher in biopsy samples from patients with severe IBD compared to those with mild disease [58]. DSS induction led to an increased abundance of Pseudomonadota (Figure 9D), which is consistent with the findings of Xu et al. [59]. However, intervention with different doses of PAE reduced Pseudomonadota abundance by 44.09%, 37.25%, and 85.90%, respectively. These results indicate that, at the phylum level, the PAE intervention in DSS-induced colitis mice significantly reduces the Bacillota/Bacteroidota ratio and suppresses the growth of the pathogenic bacteria Pseudomonadota (*p* < 0.05).

At the genus level, *Escherichia*-*Shigella*, *Clostridium*, and *Romboutsia* were most abundant in the guts of the DSS-treated mice. Following the PAE intervention, the abundance of these pathogenic bacteria showed a decreasing trend. Particularly, the high-dose PAE treatment led to statistically significant reductions in these bacteria (*p* < 0.05) (Figure 9E–G). Among them, *Escherichia*-*Shigella* is a typical intestinal pathogenic bacterium capable of producing endotoxins. Excessive endotoxins can damage the intestinal barrier, and elevated abundance of this genus may affect inflammatory-related metabolic pathways, such as linoleic acid metabolism and arachidonic acid metabolism, while increasing carcinogenic risk [60]. *Clostridium* is an opportunistic pathogen. Toxigenic *Clostridium* strains secrete two glucosylating toxins, TcdA and TcdB, which can trigger inflammation and diarrhea [61]. *Romboutsia* often accumulates in the intestines of patients with irritable bowel syndrome and gastric cancer. Its abundance shows positive correlations with the expression of multiple pro-inflammatory cytokine genes, including CXCL1, CXCL9, CCL7, and TNF-α [62]. In contrast, bacterial genera such as *norank*_*f*_*Muribaculaceae*, *norank*_*o*_*Clostridia*_*UCG*-*014*, and *Lachnospiraceae*_*NK4A136*_*group* were in greater abundance in the guts of PAE-treated mice compared to DSS-treated mice (Figure 9H–J). The PAE intervention led to an increasing trend, indicating that PAE promoted the proliferation of these bacterial genera. Among them, *norank*_*f*_*Muribaculaceae* can compete with pathogenic bacteria for colonization sites and nutrients in the intestinal mucus layer, thereby resisting colonization by intestinal pathogens and helping to restore normal intestinal barrier function [63]. Reportedly, *norank*_*o*_*Clostridia*_*UCG*-*014* shows a negative correlation with arachidonic acid metabolism levels. Therefore, an increase in its abundance can alleviate inflammatory responses during intestinal barrier repair [64]. Meanwhile, *Lachnospiraceae*_*NK4A136*_*group* is an SCFA-producing bacterium capable of decomposing dietary components, such as polyphenols and polysaccharides, to produce SCFAs, thereby maintaining normal intestinal barrier function [65]. In summary, these results demonstrate that PAE intervention suppresses the accumulation of pathogenic microorganisms in the gut while promoting the proliferation of beneficial bacterial genera, significantly ameliorating DSS-induced gut microbiota dysbiosis in mice.

### 3.9. Effect of PAE on SCFA Content in Cecal

**Figure 10:**
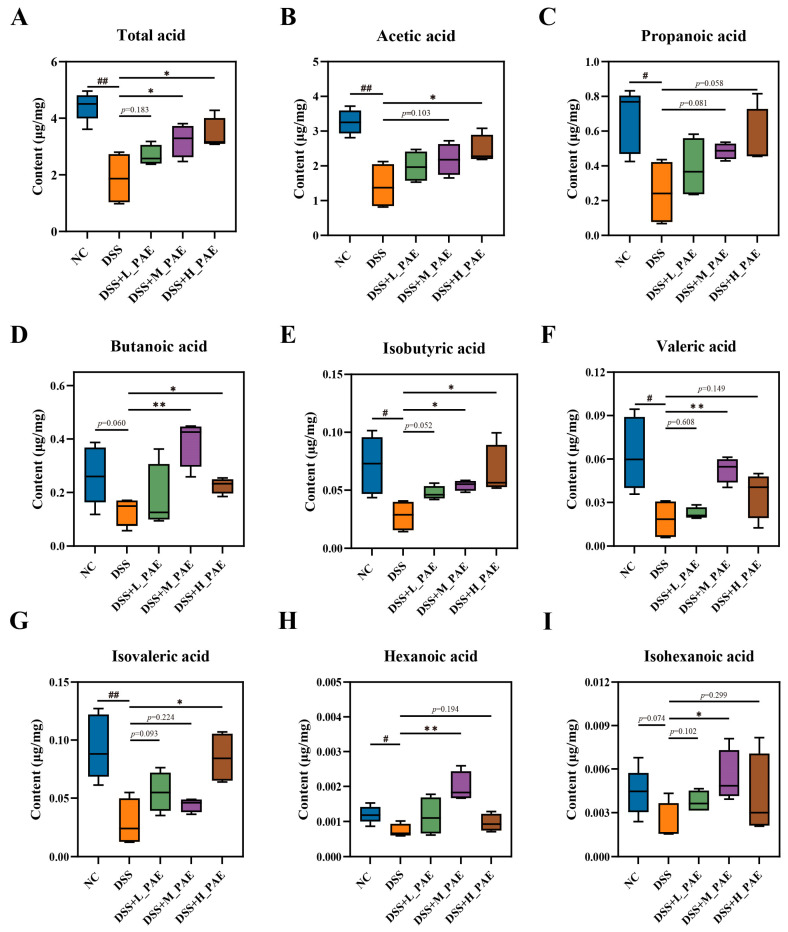
SCFA content in the cecal contents of each group of mice. (**A**) Total acid; (**B**) acetic acid; (**C**) propanoic acid; (**D**) butanoic acid; (**E**) isobutyric acid; (**F**) valeric acid; (**G**) isovaleric acid; (**H**) hexanoic acid; (**I**) isohexanoic acid. Data are presented as mean ± SEM. Analysis was carried out using one-way analysis of variance (ANOVA) (compared with NC group, # *p* < 0.05, ## *p* < 0.01; compared with DSS group, * *p* < 0.05, ** *p* < 0.01).

SCFAs are important metabolites produced by gut microbiota during the fermentation of indigestible dietary components, and they possess multiple physiological functions that promote intestinal health [25,26]. Moreover, the content of SCFAs in the intestine is closely associated with the structure of gut microbiota [66]. To analyze the effect of the PAE treatment on SCFA levels in mouse cecal contents, a quantitative analysis of SCFAs was performed in the cecal contents of each group of mice. As shown in Figure 10, the contents of total acids, acetic acid, propanoic acid, and butanoic acid in the cecal contents of the DSS-treated mice were significantly lower compared to the NC group (*p* < 0.05). This may be due to the disruption of the normal gut microbiota structure in mice by DSS, which caused reduced relative abundance of SCFA-producing bacteria. The total acid content change in mouse cecum is shown in Figure 10A. The total acid content sharply decreased from 4.42 ± 0.23 μg/mg in the NC group to 1.55 ± 0.49 μg/mg in the DSS group. Treatment with different doses of PAE restored the total acid levels to 2.24 ± 0.46 μg/mg, 2.66 ± 0.59 μg/mg, and 2.86 ± 0.61 μg/mg, respectively. Furthermore, acetic acid, propanoic acid, and butanoic acid dominate the total SCFA composition. Among them, acetic acid is the predominant SCFA in the human intestine and exhibits anti-inflammatory activity. It can also suppress appetite, influence energy expenditure, and modulate lipid metabolism [67]. Therefore, its concentration impacts the progression of intestinal inflammation. The acetic acid content is shown in Figure 10B. The acetic acid levels in the DSS group were 1.42 ± 0.32 μg/mg, significantly lower than 3.26 ± 0.16 μg/mg in the NC group (*p* < 0.05). The PAE intervention restored it to 1.98 ± 0.22 μg/mg (DSS + L_PAE group), 2.18 ± 0.23 μg/mg (DSS + M_PAE group), and 2.46 ± 0.21 μg/mg (DSS + H_PAE group). Additionally, propanoic acid can act on lipoxygenase, PPARγ receptors, GPR41, and GPR43 to inhibit the transcription factor NF-κB, thereby elevating the threshold for inflammatory responses and exerting anti-inflammatory effects [68]. The propanoic acid content in the DSS group decreased sharply to 0.25 ± 0.09 μg/mg compared to the NC group. The PAE treatment significantly restored it to 0.39 ± 0.09 μg/mg (DSS + L_PAE group), 0.48 ± 0.02 μg/mg (DSS + M_PAE group), and 0.55 ± 0.09 μg/mg (DSS + H_PAE group) (*p* < 0.05) (Figure 10C). Butyrate participates in the tricarboxylic acid cycle and serves as an energy source, providing approximately 70% of the energy for colonic epithelial cells [69]. Therefore, an increase in its concentration helps to maintain intestinal barrier integrity. The PAE treatment significantly reversed this trend, increasing butanoic acid content from 0.13 ± 0.03 μg/mg (DSS group) to 0.18 ± 0.06 μg/mg (DSS + L_PAE group), 0.39 ± 0.04 μg/mg (DSS + M_PAE group), and 0.23 ± 0.01 μg/mg (DSS + H_PAE) (Figure 10D,E). According to the LEfSe analysis (Figure 8E), the dominant bacterial genus in the DSS + M_PAE group was *Roseburia*, a butyrate-producing bacterium, which could explain the higher butanoic acid content compared to the DSS + H_PAE group. In addition, valeric acid and hexanoic acid showed an upward trend in mice after PAE supplementation (Figure 10F–I). A previous study showed that valeric acid is significantly negatively correlated with UC disease severity, monocyte count, and IL-6 levels, and supplementation with valeric acid can reduce IL-6 production [70]. Reportedly, the concentration of hexanoic acid shows a negative correlation with disease severity in patients with Crohn’s disease [71], which suggests a mitigating effect on intestinal inflammation. In summary, these results indicate that PAE promotes the production of SCFAs, including acetic acid, propanoic acid, butanoic acid, isobutyric acid, valeric acid, isovaleric acid, hexanoic acid, and isohexanoic acid, in the mouse intestine, thereby alleviating DSS-indueced colitis through improvements in intestinal barrier function and anti-inflammatory effects.

### 3.10. Correlation Analysis

**Figure 11:**
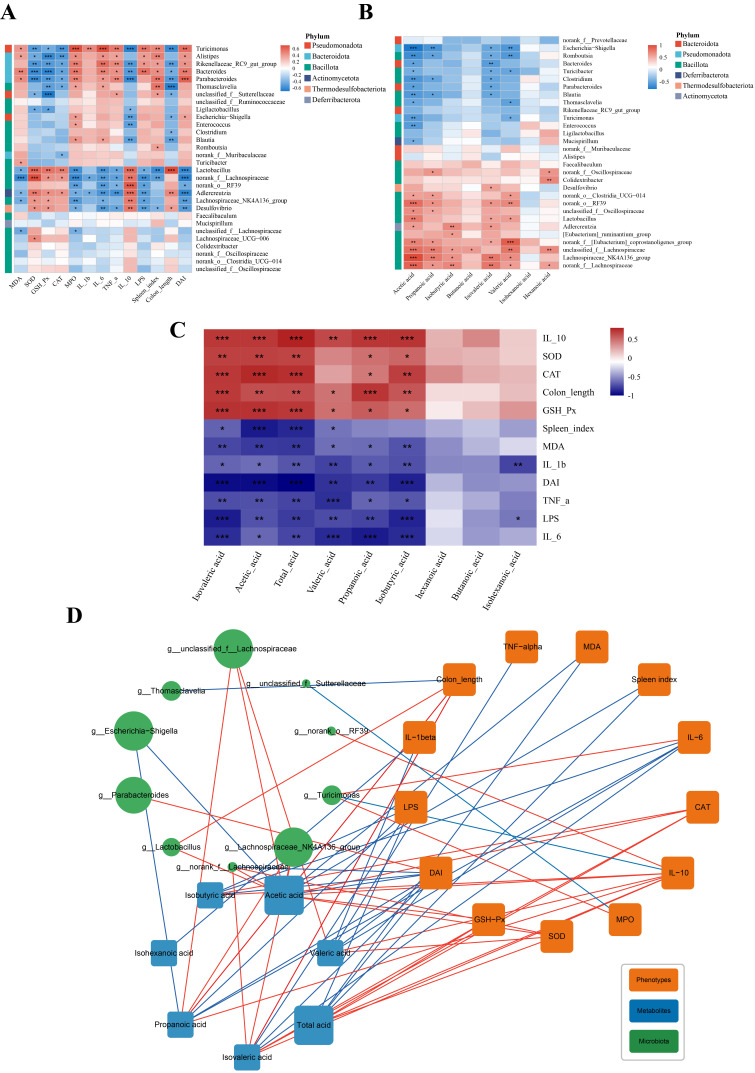
Correlation analysis of gut microbiota, SCFAs, and colitis-associated phenotypes. (**A**) Heatmap of Spearman’s correlations between phenotypic parameters and gut microbiota; (**B**) SCFAs and gut microbiota; (**C**) SCFAs and phenotypic parameters, * represents *p* < 0.05, ** represents *p* < 0.01, *** represents *p* < 0.001; (**D**) network constructed from gut microbiota, SCFAs, and colitis-associated phenotypes. The network is shown with correlation *r* > |0.6| and *p* < 0.05. Green nodes represent gut microbiota. Blue nodes represent SCFAs. Orange nodes represent colitis-associated phenotypes. The color of edges between nodes indicates the positive (red) and negative (blue) correlations. The width of the edge represents the strength of correlation. The node size represents the number of associations.

A correlation analysis provides a clear and intuitive way to visualize the relationships between two or more variables. Correlation analyses were performed to elucidate the potential associations among pathological indicators, SCFAs, and key bacterial genera in gut microbiota of mice. Correlation analyses were performed between the gut microbiota and colitis pathological phenotype indicators (Figure 11A), between the gut microbiota and SCFA levels (Figure 11B), and between the SCFA levels and colitis pathological phenotype indicators (Figure 11C). Additionally, an integrated correlation network analysis of the three components (Figure 11D) was conducted.

The Spearman correlation analysis revealed that bacterial genera such as *Turicimonas*, *Alistipes*, *Rikenellaceae*_*RC9*_*gut*_*group*, *Bacteroides*, and *Parabacteroides* showed positive correlations with indicators including DAI score, spleen index, and inflammatory mediators (MDA, TNF-α, IL-6, IL-1β, MPO, and LPS) while displaying negative correlations with antioxidant and anti-inflammatory indicators such as SOD, GSH-Px, and IL-10 (Figure 11A). This suggests that an increase in the abundance of these bacterial genera may exacerbate oxidative stress and inflammatory responses in mice. *Turicinonas* is an intestinal pathogen. High abundance of *Alistipes* can exacerbate colitis symptoms [72]. Bacteroides can induce massive recruitment of inflammatory cells to the intestinal mucosa, increasing mucosal permeability [73]. *Parabacteroides* is significantly enriched in the intestines of patients with Crohn’s disease [74], indicating that its elevated abundance may aggravate the condition. Beneficial gut bacteria, including *Desulfovibrio*, *Adlercreutzia*, *Lactobacillus*, *Lachnospiraceae*_*NK4A136*_*group*, and *norank*_*f*_*Lachnospiraceae*, showed significant negative correlations with DAI score, spleen index, inflammatory mediators (TNF-α, IL-6, and IL-1β), and intestinal permeability (reflected by serum LPS levels). Among them, it is worth noting that *Desulfovibrio* is an opportunistic pathogen, which may sometimes promote host health while also inducing the onset and progression of disease. In this study, *Desulfovibrio* showed significant positive correlations (*p* < 0.05) with anti-inflammatory (IL-10) and antioxidant (SOD and GSH-Px) indicators and was abundantly present in normal mice. This suggests that *Desulfovibrio* may be a key bacterium in ameliorating colitis, which is consistent with a previous report [75]. *Adlercreutzia* is a beneficial gut bacterium involved in the metabolism of dietary flavonoids, such as catechins [76]. *Lactobacillus* is a typical beneficial intestinal bacterium that exhibits antagonistic effects against gut pathogens, such as *Staphylococcus aureus* and *Enterococcus faecalis* [77]. Additionally, a correlation analysis was conducted between dominant gut bacterial genera and the content of SCFAs in the cecum, with the results presented in Figure 11B. Bacterial genera such as *norank*_*f*_*Lachnospiraceae*, *Lachnospiraceae*_*NK4A136*_*group*, *unclassified*_*f*_*Lachnospiraceae*, and *norank*_*f*_[*Eubacterium*]_*coprostanoligenes*_*group* showed significant positive correlations with multiple SCFAs (*p* < 0.05). Previous studies have reported that *Lachnospiraceae*_*NK4A136*_*group* and *norank*_*f*_[*Eubacterium*]_*coprostanoligenes* group are capable of producing butyrate [65]. *Unclassified*_*f*_*Lachnospiraceae* can produce SCFAs involved in anti-inflammatory effects [78]. These bacterial genera have also been shown to exhibit negative correlations with disease parameters [78]. Furthermore, a Spearman correlation analysis was performed between SCFA levels and colitis pathological indicators (Figure 11C). The results reveal that the SCFA levels showed positive correlations with the anti-inflammatory cytokine (IL-10), antioxidant enzyme activities (SOD, CAT, and GSH-Px), and colon length while exhibiting negative correlations with pro-inflammatory cytokines (IL-6, TNF-α, and IL-1β), LPS, MDA, spleen index, and DAI score. Finally, a correlation network diagram (Figure 11D) was constructed to comprehensively illustrate the interrelationships among colitis pathological parameters, gut microbiota, and SCFAs. In summary, the above results indicate that the PAE intervention modulated the composition of the gut microbiota, suppressed the proliferation of pro-inflammatory and pro-oxidative bacteria, and promoted the growth of SCFA-producing bacterial genera. Therefore, it is further confirmed that PAE alleviates DSS-induced colitis in mice by regulating gut microbial balance and promoting SCFA production.

Taken together, the above findings suggest that PAE exerts a protective effect against DSS-induced colitis. However, several limitations of this study should be acknowledged. Because PAE was administered before and during DSS exposure, the present experimental design cannot fully distinguish whether the observed effects are attributable to protection against DSS-induced epithelial injury, suppression of subsequent inflammatory progression, or both. Further studies using post-induction treatment protocols and chronic/relapsing colitis models are needed to evaluate the therapeutic efficacy of PAE more independently.

## 4. Conclusions

In summary, PAE exerts a mitigating effect on DSS-induced colitis in mice. Our findings demonstrate that PAE alleviates pathological symptoms and intestinal damage in colitis-affected mice. PAE not only inhibits pro-inflammatory cytokines, LPS secretion, and MPO activity but also mitigates oxidative stress damage in DSS-induced colitis mice and promotes mucus layer repair. More importantly, PAE could increase gut microbiota richness in DSS-induced colitis mice and change its composition. At the phylum level, PAE reduced the Bacillota/Bacteroidota ratio and suppressed pathogenic Pseudomonadota. A genus-level analysis revealed that PAE promoted the abundance of beneficial genera (*norank_f*_*Muribaculaceae*, *norank_o_Clostridia*_*UCG*-*014*, and *Lachnospiraceae*_*NK4A136*_*group*) while downregulating pro-inflammatory genera (including *Escherichia-Shigella*, *Clostridium*, and *Romboutsia*). Furthermore, different doses of PAE promoted the biosynthesis of SCFAs in the gut, which is beneficial for maintaining the intestinal barrier and reducing inflammatory responses. Overall, these findings highlight the possibility of phenolamide compounds in bee pollen to prevent colitis, which may promote the application of bee pollen in the health food sector.

## Figures and Tables

**Table 1:** Mass spectrometry and purity information of the main compounds in PAE.

Peak Number	t_R_ (min)	[M + H] ^+^ (*m*/*z*)	Proposed Molecular Formula	Major Fragment Ions (*m*/*z*)	Identification	Purity (%)
1	11.043	584.2758	C_34_H_37_N_3_O_6_	584/438/292/204/147	Tri-*p*-coumaroyl spermidine ^1^	3.16 ± 0.09
2	11.867	584.2737	C_34_H_37_N_3_O_6_	584/438/292/204/147	Tri-*p*-coumaroyl spermidine ^2^	16.38 ± 0.46
3	12.763	584.2729	C_34_H_37_N_3_O_6_	584/438/292/204/147	Tri-*p*-coumaroyl spermidine ^3^	27.23 ± 0.77
4	13.863	584.2773	C_34_H_37_N_3_O_6_	584/438/292/204/147	Tri-*p*-coumaroyl spermidine ^4^	51.01 ± 1.44
Total						97.78 ± 2.76

The superscript numbers represent isomers.

## Data Availability

The original contributions presented in this study are contained in the article/Supplementary Materials. Further inquiries can be directed to the corresponding author.

## Supplementary Materials

The following supporting information can be downloaded at: https://www.mdpi.com/article/10.3390/antiox15030403/s1, Figure S1: Animal experiment design diagram; Figure S2: Total ion chromatography (TIC) of SCFAs in representative samples from each group; Table S1: DAI scoring criteria in mice; Table S2: Histology scoring criteria in mice; Table S3: Calibration curves, correlation coefficients, and linear ranges for 8 analytes.

## References

[1] Alatab S., Sepanlou S.G., Ikuta K., Vahedi H., Bisignano C., Safiri S., Sadeghi A., Nixon M.R., Abdoli A., Abolhassani H. (2020). The global, regional, and national burden of inflammatory bowel disease in 195 countries and territories, 1990–2017: A systematic analysis for the Global Burden of Disease Study 2017. Lancet Gastroenterol. Hepatol..

[2] Windsor J.W., Kaplan G.G. (2019). Evolving epidemiology of IBD. Curr. Gastroenterol. Rep..

[3] Lee M., Chang E.B. (2021). Inflammatory bowel diseases (IBD) and the microbiome—Searching the crime scene for clues. Gastroenterology.

[4] Rogler G. (2010). Gastrointestinal and liver adverse effects of drugs used for treating IBD. Best Pract. Res. Clin. Gastroenterol..

[5] Zhang Y., Liu Y., Luo J., Liu Y., Yu S., Liu J. (2023). *Rheum tanguticum* polysaccharide alleviates DSS-induced ulcerative colitis and regulates intestinal microbiota in mice. Food Biosci..

[6] Lu S., Na K., Wei J., Tao T., Zhang L., Fang Y., Li X., Guo X. (2023). Alginate oligosaccharide structures differentially affect DSS-induced colitis in mice by modulating gut microbiota. Carbohydr. Polym..

[7] Zhou S., Yang J., Pan Y., Feng X., Hu H., Ma S., Ou C., Fan F., Gong S., Wang Y. (2023). Pu’ er raw tea extract alleviates DSS-induced colitis in mice by restoring intestinal barrier function and maintaining gut microbiota homeostasis. Food Biosci..

[8] Wang N., Chen W., Cui C., Zheng Y., Yu Q., Ren H., Liu Z., Xu C., Zhang G. (2022). The peanut skin procyanidins attenuate DSS-induced ulcerative colitis in C57BL/6 mice. Antioxidants.

[9] Ning K., Duan Y., Tong W., Chen Y., Zhang Q., Xie Q., Xiang H. (2023). Protective effects of different molecular weights of purslane (*Portulaca oleracea* L.) aqueous extract on DSS-induced ulcerative colitis in mice. Antioxidants.

[10] Anjum S.I., Ullah A., Gohar F., Raza G., Khan M.I., Hameed M., Ali A., Chen C.-C., Tlak Gajger I. (2024). Bee pollen as a food and feed supplement and a therapeutic remedy: Recent trends in nanotechnology. Front. Nutr..

[11] Thakur M., Nanda V. (2020). Composition and functionality of bee pollen: A review. Trends Food Sci. Technol..

[12] Qiao J., Feng Z., Zhang Y., Xiao X., Dong J., Haubruge E., Zhang H. (2023). Phenolamide and flavonoid glycoside profiles of 20 types of monofloral bee pollen. Food Chem..

[13] Xiong W., Li Y., Yao Y., Xu Q., Wang L. (2022). Antioxidant mechanism of a newly found phenolic compound from *adlay* (NDPS) in HepG2 cells via Nrf2 signalling. Food Chem..

[14] Sartori A.G.D., Saliba A., Martarello N.S., Lazarini J.G., do Amaral J., da Luz C.F.P., de Alencar S.M. (2024). Changes in phenolic profile and anti-inflammatory activity of *Baccharis* beebread during gastrointestinal digestion/intestinal permeability in vitro. Food Chem..

[15] Kyselka J., Bleha R., Dragoun M., Bialasová K., Horáčková Š., Schätz M., Sluková M., Filip V., Synytsya A. (2018). Antifungal polyamides of hydroxycinnamic acids from sunflower bee pollen. J. Agric. Food Chem..

[16] Zhou Q., Wang L., Liu B., Xiao J., Cheng K.-W., Chen F., Wang M. (2021). Tricoumaroylspermidine from rose exhibits inhibitory activity against ethanol-induced apoptosis in HepG2 cells. Food Funct..

[17] Zhang X., Wu X., Xiao G., Liu G., Dong H., Liu R., Lu Q. (2023). Phenolamide extract of apricot bee pollen alleviates glucolipid metabolic disorders and modulates the gut microbiota and metabolites in high-fat diet-induced obese mice. Food Funct..

[18] Zhang X., Yu M., Zhu X., Liu R., Lu Q. (2022). Metabolomics reveals that phenolamides are the main chemical components contributing to the anti-tyrosinase activity of bee pollen. Food Chem..

[19] Qiao J., Cai W., Wang K., Eric H., Dong J., Hesham R.E.-S., Xu X., Zhang H. (2024). New insights into identification, distribution, and health benefits of polyamines and their derivatives. J. Agric. Food Chem..

[20] Liu H., Liu Y., Han H., Lu C., Chen H., Chai Y. (2023). Identification and characterization of phenolamides in tea (*Camellia sinensis*) flowers using ultra-high-performance liquid chromatography/Q-Exactive orbitrap mass spectrometry. Food Chem..

[21] Qiao J., Zhang Y., Haubruge E., Wang K., El-Seedi H.R., Dong J., Xu X., Zhang H. (2024). New insights into bee pollen: Nutrients, phytochemicals, functions and wall-disruption. Food Res. Int..

[22] Chen J., Xiao Y., Li D., Zhang S., Wu Y., Zhang Q., Bai W. (2023). New insights into the mechanisms of high-fat diet mediated gut microbiota in chronic diseases. iMeta.

[23] Jin S., Zhao D., Cai C., Song D., Shen J., Xu A., Qiao Y., Ran Z., Zheng Q. (2017). Low-dose penicillin exposure in early life decreases Th17 and the susceptibility to DSS colitis in mice through gut microbiota modification. Sci. Rep..

[24] Scaldaferri F., D’Onofrio A.M., Calia R., Di Vincenzo F., Ferrajoli G.F., Petito V., Maggio E., Pafundi P.C., Napolitano D., Masi L. (2023). Gut microbiota signatures are associated with psychopathological profiles in patients with ulcerative colitis: Results from an Italian tertiary IBD center. Inflamm. Bowel Dis..

[25] Chen T., Kim C.Y., Kaur A., Lamothe L., Shaikh M., Keshavarzian A., Hamaker B.R. (2017). Dietary fibre-based SCFA mixtures promote both protection and repair of intestinal epithelial barrier function in a caco-2 cell model. Food Funct..

[26] Li M., van Esch B.C.A.M., Wagenaar G.T.M., Garssen J., Folkerts G., Henricks P.A.J. (2018). Pro- and anti-inflammatory effects of short chain fatty acids on immune and endothelial cells. Eur. J. Pharmacol..

[27] Taladrid D., de Llano D.G., Zorraquín-Peña I., Tamargo A., Silva M., Molinero N., Moreno-Arribas M.V., Bartolomé B. (2021). Gastrointestinal digestion of a grape pomace extract: Impact on intestinal barrier permeability and interaction with gut microbiome. Nutrients.

[28] Huang G., Wang Z., Wu G., Zhang R., Dong L., Huang F., Zhang M., Su D. (2021). Lychee (*Litchi chinensis* sonn.) pulp phenolics activate the short-chain fatty acid-free fatty acid receptor anti-inflammatory pathway by regulating microbiota and mitigate intestinal barrier damage in dextran sulfate sodium-induced colitis in mice. J. Agric. Food Chem..

[29] Xia P., Hou T., Ma M., Li S., Jin H., Luo X., Li J., Geng F., Li B. (2022). Konjac oligosaccharides attenuate DSS-induced ulcerative colitis in mice: Mechanistic insights. Food Funct..

[30] Guo B., Zhang W., Zhang J., Zou J., Dong N., Liu B. (2025). *Euglena gracilis* polysaccharide modulated gut dysbiosis of obese individuals via acetic acid in an in vitro fermentation model. Food Res. Int..

[31] Yang M., Tao L., Wang Z., Li L., Zhao C., Shi C., Sheng J., Tian Y. (2024). Effects of UV/H_2_O_2_ degradation on *Moringa oleifera* Lam. leaves polysaccharides: Composition, in vitro fermentation and prebiotic properties on gut microorganisms. Food Chem. X.

[32] Dong P., Ge G., Zhang Y., Ai C., Li G., Zhu L., Luan H., Liu X., Yang L. (2009). Quantitative structure-retention relationship studies for taxanes including epimers and isomeric metabolites in ultra fast liquid chromatography. J. Chromatogr. A.

[33] Jain P., Hassan A.M., Koyani C.N., Mayerhofer R., Reichmann F., Farzi A., Schuligoi R., Malle E., Holzer P. (2015). Behavioral and molecular processing of visceral pain in the brain of mice: Impact of colitis and psychological stress. Front. Behav. Neurosci..

[34] Melgar S., Bjursell M., Gerdin A.K., Svensson L., Michaëlsson E., Bohlooly-Y M. (2007). Mice with experimental colitis show an altered metabolism with decreased metabolic rate. Am. J. Physiol.-Gastrointest. Liver Physiol..

[35] Shafiee N.H., Manaf Z.A., Mokhtar N.M., Raja Ali R.A. (2020). An assessment of dietary intake, food avoidance and food beliefs in patients with ulcerative colitis of different disease status. Intest. Res..

[36] Wen C., Hu H., Yang W., Zhao Y., Zheng L., Jiang X., Wang L. (2022). Targeted inhibition of FcRn reduces NET formation to ameliorate experimental ulcerative colitis by accelerating ANCA clearance. Int. Immunopharmacol..

[37] Lewis S.M., Williams A., Eisenbarth S.C. (2019). Structure and function of the immune system in the spleen. Sci. Immunol..

[38] Jin Y., Wu J., Huang K., Liang Z. (2024). Heat-killed *Saccharomyces boulardii* alleviates dextran sulfate sodium-Induced ulcerative colitis by restoring the intestinal barrier, reducing inflammation, and modulating the gut microbiota. Nutrients.

[39] Abd El-Aziz R., Naguib M., Rashed L.A. (2018). Spleen size in patients with metabolic syndrome and its relation to metabolic and inflammatory parameters. Egypt. J. Intern. Med..

[40] Wang A., Yang X., Lin J., Wang Y., Yang J., Zhang Y., Tian Y., Dong H., Zhang Z., Song R. (2025). Si-Ni-San alleviates intestinal and liver damage in ulcerative colitis mice by regulating cholesterol metabolism. J. Ethnopharmacol..

[41] Birben E., Sahiner U.M., Sackesen C., Erzurum S., Kalayci O. (2012). Oxidative stress and antioxidant defense. World Allergy Organ. J..

[42] Pisoschi A.M., Pop A., Iordache F., Stanca L., Predoi G., Serban A.I. (2021). Oxidative stress mitigation by antioxidants-an overview on their chemistry and influences on health status. Eur. J. Med. Chem..

[43] Vieira S., Zhang G.D., Decker E.A. (2017). Biological implications of lipid oxidation products. J. Am. Oil Chem. Soc..

[44] Limón-Pacheco J., Gonsebatt M.E. (2009). The role of antioxidants and antioxidant-related enzymes in protective responses to environmentally induced oxidative stress. Mutat. Res./Genet. Toxicol. Environ. Mutagen..

[45] Ali S.S., Ahsan H., Zia M.K., Siddiqui T., Khan F.H. (2020). Understanding oxidants and antioxidants: Classical team with new players. J. Food Biochem..

[46] Tian T., Wang Z., Zhang J. (2017). Pathomechanisms of oxidative stress in inflammatory bowel disease and potential antioxidant therapies. Oxidative Med. Cell. Longev..

[47] Xiao Y., Yan W., Cao Y., Yan J., Cai W. (2016). Neutralization of IL-6 and TNF-α ameliorates intestinal permeability in DSS-induced colitis. Cytokine.

[48] Li Y., Jia Y., Cui T., Zhang J. (2021). IL-6/STAT3 signaling pathway regulates the proliferation and damage of intestinal epithelial cells in patients with ulcerative colitis via H3K27ac. Exp. Ther. Med..

[49] Lin W., Chen H., Chen X., Guo C. (2024). The roles of neutrophil-derived myeloperoxidase (MPO) in diseases: The new progress. Antioxidants.

[50] Kong Q., Lv Z., Kang Y., An Y., Liu Z., Zhang J. (2021). Bactericidal permeability increasing protein deficiency aggravates acute colitis in mice by increasing the serum levels of lipopolysaccharide. Front. Immunol..

[51] Gu Y., Qin X., Zhou G., Wang C., Mu C., Liu X., Zhong W., Xu X., Wang B., Jiang K. (2022). *Lactobacillus rhamnosus* GG supernatant promotes intestinal mucin production through regulating 5-HT4R and gut microbiota. Food Funct..

[52] Pang B., Jin H., Liao N., Li J., Jiang C., Shi J. (2021). Vitamin A supplementation ameliorates ulcerative colitis in gut microbiota-dependent manner. Food Res. Int..

[53] Boland K., Bedrani L., Turpin W., Kabakchiev B., Stempak J., Borowski K., Nguyen G., Steinhart A.H., Smith M., Croitoru K. (2021). Persistent darrhea in patients with Crohn’s disease after mucosal healing is associated with lower diversity of the intestinal microbiome and increased dysbiosis. Clin. Gastroenterol. Hepatol..

[54] Li C., Pan J., Sun P., Wang S., Wang S., Feng W., Chen S., Chai X., Zhao S., Zhu X. (2023). Ketogenic diet alleviates hypoglycemia-induced neuroinflammation via modulation the gut microbiota in mice. Mol. Nutr. Food Res..

[55] Tsai Y.C., Tai W.C., Liang C.M., Wu C.K., Tsai M.C., Hu W.H., Huang P.Y., Chen C.H., Kuo Y.H., Yao C.C. (2025). Alternations of the gut microbiota and the firmicutes/bacteroidetes ratio after biologic treatment in inflammatory bowel disease. J. Microbiol. Immunol..

[56] Li C., Deng L., Pu M., Ye X., Lu Q. (2024). Coptisine alleviates colitis through modulating gut microbiota and inhibiting TXNIP/NLRP3 inflammasome. J. Ethnopharmacol..

[57] Miao S., Lu Q., Zhou Y., Chang Y., Xu T., Zhu M. (2022). Oral administration of octacosanol modulates the gut bacteria and protects the intestinal barrier in ulcerative colitis mice. J. Food Biochem..

[58] Acar C., Celik S.K., Ozdemirel H.O., Tuncdemir B.E., Alan S., Mergen H. (2024). Composition of the colon microbiota in the individuals with inflammatory bowel disease and colon cancer. Folia Microbiol..

[59] Xu Z., Mi S., Chitrakar B., Wang L., Li Y., Shi R., Sang Y., Yu W., Wang X. (2025). Study on the role of the combination of quercetin and lutein in alleviating ulcerative colitis in mice. Front. Nutr..

[60] Tang Q., Cang S., Jiao J., Rong W., Xu H., Bi K., Li Q., Liu R. (2020). Integrated study of metabolomics and gut metabolic activity from ulcerative colitis to colorectal cancer: The combined action of disordered gut microbiota and linoleic acid metabolic pathway might fuel cancer. J. Chromatogr. A.

[61] Anjuwon-Foster B.R., Tamayo R. (2018). Phase variation of *clostridium difficile* virulence factors. Gut Microbes.

[62] Wang H., Zhang M., Wen X., He L., Zhang M., Zhang J., Yang X. (2022). Cepharanthine ameliorates dextran sulphate sodium-induced colitis through modulating gut microbiota. Microb. Biotechnol..

[63] Zhang C., Shu Y., Li Y., Wang F., Gan J., Wang Y., Feng X., Guo M. (2025). Chinese yam (*Dioscorea*) polysaccharide ameliorates ulcerative colitis in mice via modulating disorders of intestinal microecology and metabolism. Int. J. Biol. Macromol..

[64] Gong H., Gan X., Qin B., Chen J., Zhao Y., Qiu B., Chen W., Yu Y., Shi S., Li T. (2024). Structural characteristics of steamed *polygonatum cyrtonema* polysaccharide and its bioactivity on colitis via improving the intestinal barrier and modifying the gut microbiota. Carbohydr. Polym..

[65] Liu X., Zhang Y., Li W., Zhang B., Yin J., Liuqi S., Wang J., Peng B., Wang S. (2022). Fucoidan ameliorated dextran sulfate sodium-induced ulcerative colitis by modulating gut microbiota and bile acid metabolism. J. Agric. Food Chem..

[66] Qiao B., Liu J., Peng X., Cai Y., Peng M., Li X., Tan Z., Deng N. (2023). Association of short-chain fatty acids with gut microbiota and lipid metabolism in mice with diarrhea induced by high-fat diet in a fatigued state. Mol. Nutr. Food Res..

[67] Guo C., Wang Y., Zhang S., Zhang X., Du Z., Li M., Ding K. (2021). *Crataegus pinnatifida* polysaccharide alleviates colitis via modulation of gut microbiota and SCFAs metabolism. Int. J. Biol. Macromol..

[68] Al-Lahham S.H., Peppelenbosch M.P., Roelofsen H., Vonk R.J., Venema K. (2010). Biological effects of propionic acid in humans; metabolism, potential applications and underlying mechanisms. Biochim. Biophys. Acta (BBA)-Mol. Cell Biol. Lipids.

[69] Singh N., Gurav A., Sivaprakasam S., Brady E., Padia R., Shi H., Thangaraju M., Prasad P.D., Manicassamy S., Munn D.H. (2014). Activation of gpr109a, receptor for niacin and the commensal metabolite butyrate, suppresses colonic inflammation and carcinogenesis. Immunity.

[70] Liu M., Zhang Y., Liu J., Xiang C., Lu Q., Lu H., Yang T., Wang X., Zhang Q., Fan C. (2024). Revisiting the role of valeric acid in manipulating ulcerative colitis. Inflamm. Bowel Dis..

[71] De Preter V., Machiels K., Joossens M., Arijs I., Matthys C., Vermeire S., Rutgeerts P., Verbeke K. (2015). Faecal metabolite profiling identifies medium-chain fatty acids as discriminating compounds in IBD. Gut.

[72] Niu M., He Q., Chen Y., Liu S., Pan Y., Zhu X., Miao Y., Du Y. (2025). *Alistipes putredinis* CCUG 45780^T^ exacerbates DSS-induced colitis in mice via modulation of gut microbiota and succinate metabolism. Bmc Microbiol..

[73] Basha O.M., Hafez R.A., Salem S.M., Anis R.H., Hanafy A.S. (2023). Impact of gut microbiome alteration in ulcerative colitis patients on disease severity and outcome. Clin. Exp. Med..

[74] Lopetuso L.R., Petito V., Graziani C., Schiavoni E., Sterbini F.P., Poscia A., Gaetani E., Franceschi F., Cammarota G., Sanguinetti M. (2017). Gut microbiota in health, diverticular disease, irritable bowel syndrome, and inflammatory bowel diseases: Time for microbial marker of gastrointestinal disorders?. Digest. Dis..

[75] Chen S., Wang J., Dong N., Fang Q., Zhang Y., Chen C., Cui S.W., Nie S. (2023). Polysaccharides from natural *cordyceps sinensis* attenuated dextran sodium sulfate-induced colitis in C57BL/6J mice. Food Funct..

[76] Takagaki A., Nanjo F. (2016). Biotransformation of (−)-epicatechin, (+)-epicatechin, (−)-catechin, and (+)-catechin by intestinal bacteria involved in isoflavone metabolism. Biosci. Biotechnol. Biochem..

[77] Strahinic I., Busarcevic M., Pavlica D., Milasin J., Golic N., Topisirovic L. (2007). Molecular and biochemical characterizations of human oral lactobacilli as putative probiotic candidates. Oral Microbiol. Immun..

[78] Chen M., Zhao Y., Li S., Chang Z., Liu H., Zhang D., Wang S., Zhang X., Wang J. (2024). Maternal malic acid may ameliorate oxidative stress and inflammation in sows through modulating gut microbiota and host metabolic profiles during late pregnancy. Antioxidants.

