# Supplementary material for: Phenolamide Extract of Apricot Bee Pollen Alleviates DSS-Induced Ulcerative Colitis in Mice by Reducing Oxidative Stress, Modulating Inflammation, and Regulating Gut Microbiota
Source: Antioxidants (Basel). 2026 Mar 23;15(3):403. doi: 10.3390/antiox15030403 (PMC13024737; doi:10.3390/antiox15030403)
Supplement: Supplementary file 1 [file antioxidants-15-00403-s001.zip › antioxidants-4187715-supplementary.pdf]

Supplementary Figures

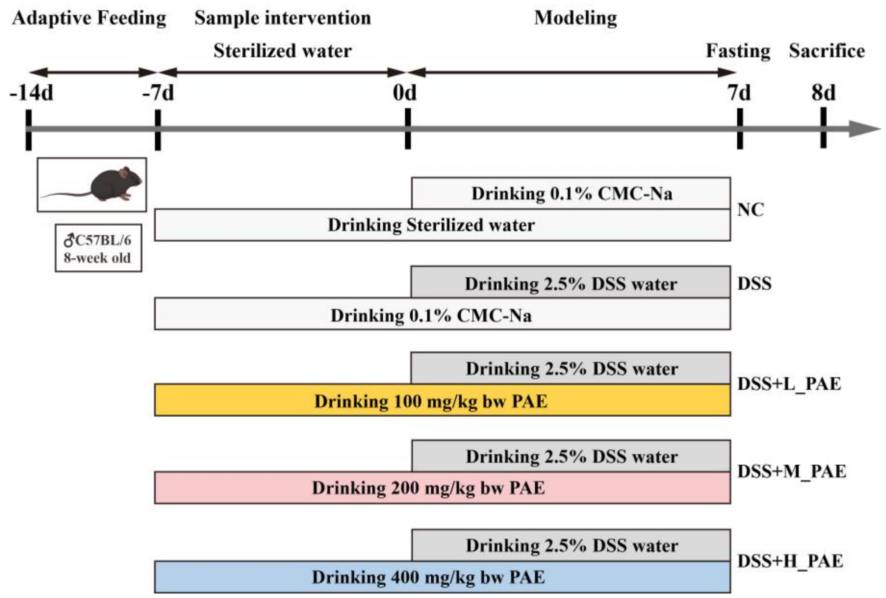

Figure S1. Animal experiment design diagram

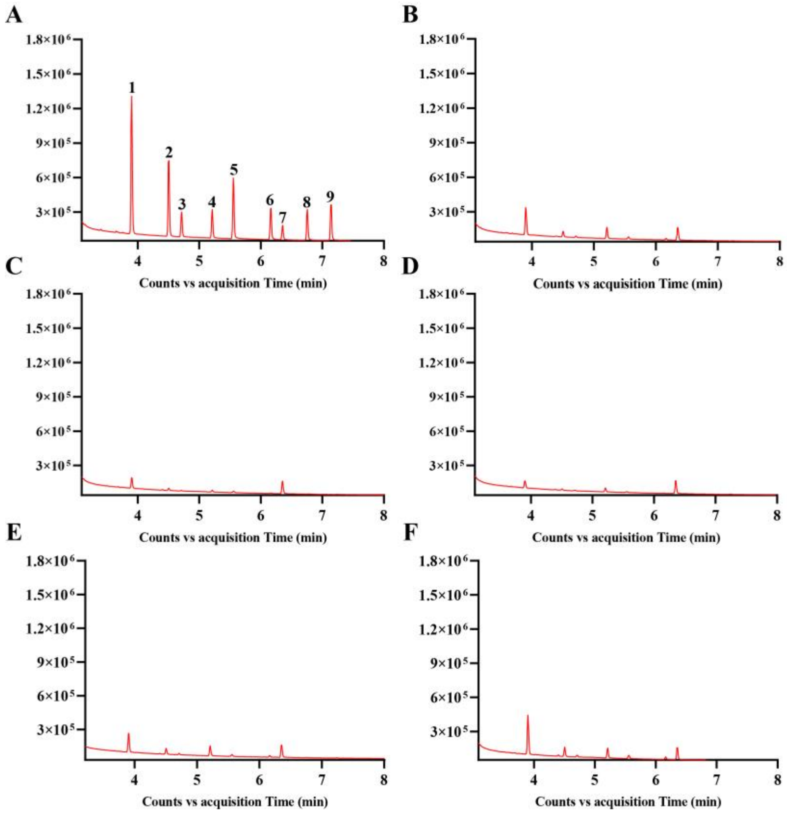

Figure S2. Total ion chromatography (TIC) of SCFAs in representative samples from each group. (A) Gas chromatograms of standard mixture; (B) Normal control group; (C) DSS-treated group; (D) DSS+L_PAE group; (E) DSS+M_PAE group; (F) DSS+H_PAE group. Peak identification: 1, acetic acid; 2, propanoic acid; 3, isobutyric acid; 4, butanoic acid; 5, isovaleric acid; 6, valeric acid; 7, 2-ethybutyric acid (internal standard); 8, isohexanoic acid; 9 hexanoic acid.

Supplementary Tables

Table S1. DAI scoring criteria in mice.

Body Weight loss	Fecal consistency	Fecal occult blood	Scores
None	Normal	Negative	0
1-5%	Soft but formed	Trace positive	1
5-10%	Soft and unformed	Positive	2
10-20%	Loose stool	Gross blood in stool	3
>20%	Severe diarrhea	Severe hematochezia	4

Table S2. Histology scoring criteria in mice.

Infiltration	Mucosa damage	Scores
None	Intact mucosa	0
Infiltrate around crypt bases	Disruption of mucosa (≤1/3)	1
Infiltrate in muscularis mucosa	Disruption of mucosa (1/3-2/3)	2
Extensive infiltrate in muscularis mucosa with edema	Disruption of mucosa (> 2/3)	3
Infiltration of the submucosa		4

Table S3. Calibration curves, correlation coefficients, and liner ranges for 8 analytes.

**Analyte**	**Calibration curves**	**R^2**	**Liner range (μg/mL)**
Acetic acid	y=0.383941x+0.007146	0.999	0.0048-600
Propanoic acid	y=0.611205x+0.003718	0.999	0.0240-240
Isobutyric acid	y=1.156864x+0.0006852	0.999	0.0040-50
Butanoic acid	y=1.803833x+0.010067	0.999	0.0040-50
Isovaleric acid	y=1.793541x+0.008698	0.999	0.0080-100
Valeric acid	y=2.147663x+0.001271	0.999	0.0040-50
Isohexanoic acid	y=0.952077x+0.005725	0.999	0.0040-50
Hexanoic acid	y=1.917762x+0.007467	0.999	0.0040-50
